# Asymmetric Synthesis of Contact Sex Pheromone of *Tetropium fuscum* and Its Enantiomer

**DOI:** 10.3390/molecules27206897

**Published:** 2022-10-14

**Authors:** Xueyang Wang, Jianan Wang, Fengbo Ma, Qinghua Bian, Min Wang, Jiangchun Zhong

**Affiliations:** 1 Department of Applied Chemistry, China Agricultural University, 2 West Yuanmingyuan Road, Beijing 100193, China; 2 College of Enviromental Sciences, Sichuan Agricultural University, 211 Huimin Road, Chengdu 611130, China

**Keywords:** contact sex pheromone, *Tetropium fuscum*, chiral auxiliary, asymmetric synthesis

## Abstract

*Tetropium fuscum* is a harmful forest pest and attacks spruces. The contact sex pheromone of this pest, (*S*)-11-methyl-heptacosane, and its enantiomer were synthesized via Evans’ chiral auxiliaries. The key steps of this approach included acylation of carboxylic acid, diastereoselective methylation of oxazolidinone amide, and Wittig coupling of the aldehyde with chiral phosphonium salt. The synthetic pheromones would have potential utility in the control of this pest.

## 1. Introduction

The brown spruce longhorn beetle, *Tetropium fuscum* Fabricius (Coleoptera: Cerambycidae), is a harmful forest pest native to Europe where it attacks weakened Norway spruce, *Picea abies* L. [1,2]. It first became invasive to North America around 1990 [3,4], where it was infecting and killing healthy native spruces, such as red spruce (*Picea rubens* Sargent), white spruce (*Picea glauca* Moench), blue spruce (*Picea pungens* Engelm), and black spruce (*Picea mariana* Miller) [5,6]. Due to its subcortical feeding habits [7,8], systemic insecticides and heating are not very effective and practical [9,10].

**Figure 1:**
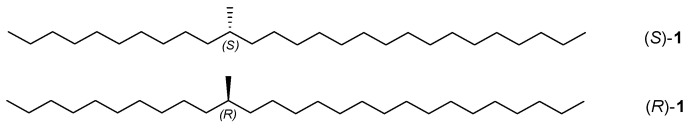
Contact sex pheromone of *Tetropium fuscum* and its enantiomer.

The strategy based on pheromones for controlling agricultural pests is one of the most promising, effective, and safe solutions [11,12]. The contact sex pheromone of *Tetropium fuscum* was identified as (*S*)-11-methyl-heptacosane ((*S*)-**1**) (Figure 1) by Silk, meanwhile, (*S*)-**1** and its enantiomer (*R*)-**1** were synthesized from (*S*)- and (*R*)-citronellyl bromides [13]. To study future utilization of the contact sex pheromone [14], herein, we prepared the contact sex pheromone of *Tetropium fuscum* and its enantiomer using Evans’ chiral auxiliaries. Our synthesis was easily performed and afforded the target pheromone with high enantiomeric purity.

## 2. Results and Discussion

### 2.1. Retrosynthetic Analysis

**Scheme 1:**
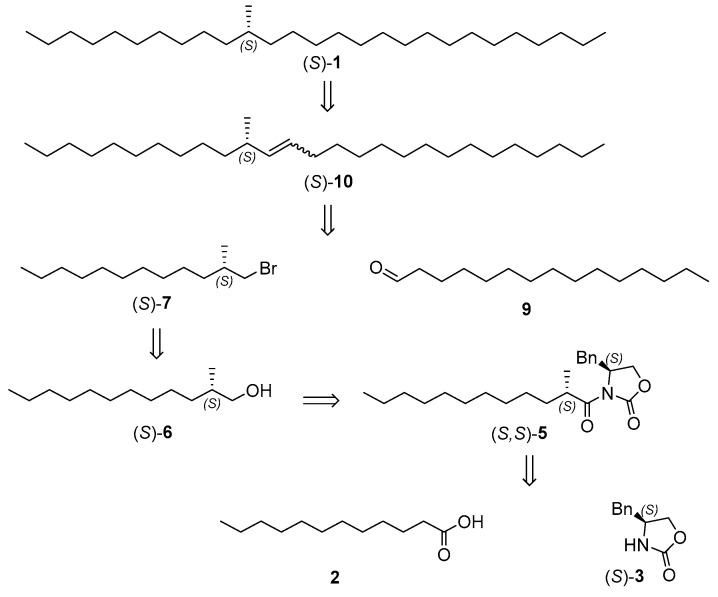
Retrosynthetic analysis of contact sex pheromone (*S*)-**1**.

In view of retrosynthetic analysis of contact sex pheromone of (*S*)-**1** (Scheme 1), the key step is to construct the chiral center. It was envisaged that Evans’ chiral auxiliaries including acylation of dodecanoic acid (**2**) and diastereoselective methylation of oxazolidinone amide would introduce chiral methyl of amide (*S*,*S*)-**5**. The target pheromone (*S*)-**1** could be synthesized via hydrogenation reduction of olefin (*S*)-**10**, which could be divided into two components, pentadecanal (**9**) and phosphonium salt in situ prepared by hydrocarbon bromide (*S*)-**7** and triphenylphosphine. Furthermore, (*S*)-1-bromo-2-methyldodecane ((*S*)-**7**) could be easily prepared from chiral alcohol (*S*)-**6** through Appel reaction.

### 2.2. Synthesis of Chiral Primary Bromides

**Scheme 2:**
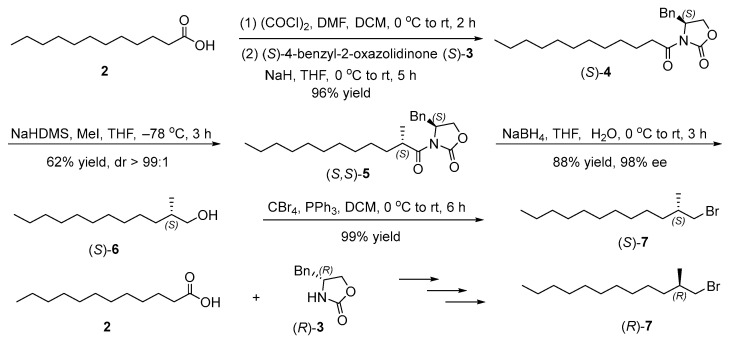
Synthesis of chiral primary bromides (*S*)- and (*R*)-**7**.

Based on the retrosynthetic analysis of contact sex pheromone (*S*)-**1**, chiral primary bromides (*S*)- and (*R*)-**7** were first prepared (Scheme 2). The reaction of dodecanoic acid (**2**) with oxalyl chloride afforded the corresponding crude acyl chloride [15] were then treated with oxazolidinone (*S*)-**3** and NaH to provide (*S*)-4-benzyl-3-dodecanoyloxazolidin-2-one ((*S*)-**4**) in a 96% yield [16]. In the presence of hexamethyldisilazide (NaHMDS), diastereoselective methylation of amide (*S*)-**4** with methyl iodide gave chiral methyl amide (*S*,*S*)-**5** (62% yield, dr > 99:1, determined by ^13^C NMR spectra) [17]. According to Evans’ chiral auxiliary precedent [18], the absolute configuration of the new stereocenter of (*S*,*S*)-**5** was assigned as (*S*). The subsequent reduction with NaBH_4_ afforded (*S*)-2-methyldodecan-1-ol ((*S*)-**6**) in an 88% yield [19,20]. Its specific rotation {[α]_D_^22^ = −9.0 (*c* 1.37, CHCl_3_)} was identical to the literature [21] value {[α]_D_^23^ = −8.4 (*c* 1.00, CHCl_3_)}, which also supported the (*S*)-configuration of the new stereocenter. The final Appel reaction converted the chiral methyl alcohol (*S*)-**6** to (*S*)-1-bromo-2-methyldodecane ((*S*)-**7**) in a 99% yield [22,23]. Similarly, (*R*)-1-bromo-2-methyldodecane ((*R*)-**7**) was prepared via acylation, diastereoselective methylation, reduction, and bromination from dodecanoic acid (**2**) and (*R*)-4-benzyloxazolidin-2-one ((*R*)-**3**).

### 2.3. Research on the Enantiomeric Purity of Chiral Alcohols

**Scheme 3:**
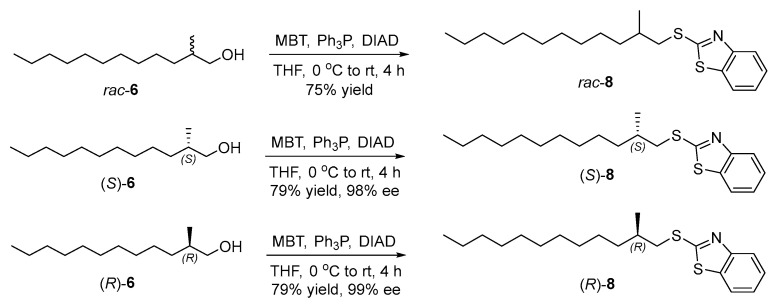
Synthesis of MBT derivatives of alcohols **6**.

To explore the optical purity of key intermediate chiral methyl alcohols (*S*)-**6** and (*R*)-**6**, we synthesized their MBT derivatives (Scheme 3). According to the similar sequence of (*S*)-***6***, the racemic alcohol **6** was prepared from dodecanoic acid and oxazolidin-2-one. Then, the Mitsunobu reaction of methyl alcohols **6** with benzo[*d*]thiazole-2(*3H*)-thione (MBT) afforded their MBT derivatives **8** [24,25]. Ee of (*S*)-**8** and (*R*)-**8** was 98–99% determined by HPLC with a Daciel Chiralcel OD-H column, which indicated that the enantiomeric purity of (*S*)-**6** and (*R*)-**6** were also 98–99%.

### 2.4. Synthesis of the Target Compounds

**Scheme 4:**
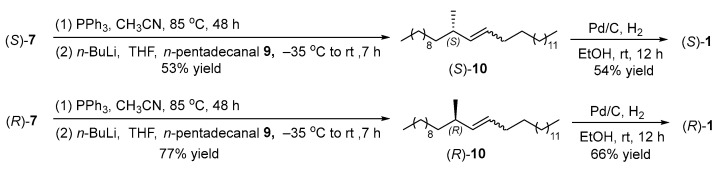
Synthesis of contact sex pheromone (*S*)-**1** and its enantiomer (*R*)-**1**.

With chiral primary bromides in hand, we focused on the synthesis of the target pheromone (*S*)-**1** and its enantiomer (*R*)-**1** (Scheme 4)**.** The *Z*/*E* mixtures of olefin (*S*)-**10** were achieved through Wittig coupling of *n*-pentadecanal (**9**) and phosphonium salt (53% yield, *Z*:*E* = 5.6:1, determined by ^13^C NMR spectra) [26,27], which was prepared in situ from hydrocarbon bromide (*S*)-**7** and triphenylphosphine[28]. The final palladium-catalyzed hydrogenation of (*S*)-**10** afforded the target compound (*S*)-11-methylheptacosane ((*S*)-**1**) [29,30]. The NMR spectrum and specific rotation of (*S*)-**1** were matched with the literature [13]. Using the similar approach of pheromone (*S*)-**1**, we synthesized its enantiomer, (*R*)-11-methylheptacosane ((*R*)-**1**). Moreover, the structure of (*R*)-**1** was characterized with ^1^H NMR, ^13^C NMR, and EIMS spectra, which were consistent with reference 13. 

## 3. Materials and Methods

### 3.1. General Information

All reactions were performed in a Schlenk system under an argon atmosphere unless otherwise indicated. All commercial reagents were used directly, whereas solvents were purified following the standard strategies before use. Polarimetric measurements were taken on a Perkin–Elmer PE-341 polarimeter. Enantiomeric excesses were determined by an Agilent 1200 HPLC system with a Daicel Chiralcel OD-H column with the eluents of *n*-hexane and isopropanol. ^1^H and ^13^C NMR spectra were recorded on a Bruker DP-X500 MHz spectrometer. Chemical shifts were reported in ppm relative to tetramethylsilane for ^1^H NMR and CDCl_3_ (77.16 ppm) for ^13^C NMR. High resolution mass spectra were collected on Waters LCT Premier™ with an ESI mass spectrometer. Low-resolution mass spectra were obtained from an Exactive GC-MS (EI).

### 3.2. Synthesis of (S)-4-Benzyl-3-dodecanoyloxazolidin-2-one ((S)-***4***) (CAS 198649-20-6)

The catalytic amount of DMF was added to a stirred solution of dodecanoic acid (**2**) (5.00 g, 24.97 mmol) in DCM (40 mL) at 0 °C. Oxalyl chloride (4.75 g, 37.43 mmol) was then added dropwise, and the reaction mixture was stirred for 1 h at 0 °C. After being warmed to room temperature and maintained for another 1 h, the solvent was removed under reduced pressure. The crude dodecanoyl chloride (3.49 g, 64% yield) was obtained as a slight yellow solid.

NaH (0.99 g, 60% in mineral oil, 24.75 mmol) was added in portions to a stirred solution of (*S*)-4-benzyloxazolidin-2-one ((*S*)-**3**) (2.95 g, 16.64 mmol, >99% ee) in THF (20 mL) at 0 °C. The resulting mixture was warmed to room temperature and stirred for 2 h, followed by the addition of the crude dodecanoyl chloride. The reaction mixture was maintained for another 3 h at the same temperature, then quenched with saturated aqueous NH_4_Cl (10 mL). After the layers were separated, the aqueous phase was extracted with EtOAc (3 × 50 mL). The EtOAc extracts were combined with organic layer, washed with saturated aqueous NaCl (50 mL), dried over anhydrous Na_2_SO_4_, and filtered. The filtrate was concentrated under reduced pressure, and the residue was purified by silica gel column chromatography (EtOAc/petroleum ether 2:8) to afford (*S*)-4-benzyl-3-dodecanoyloxazolidin-2-one ((*S*)-**4**) as a colorless oil (5.73 g, 96% yield). [α]_D_^22^ = +32.8 (c = 2.67, CHCl_3_). ^1^H NMR (500 MHz, CDCl_3_) δ 7.33–7.20 (m, 5H), 4.67 (ddt, *J* = 10.7, 7.3, 3.2 Hz, 1H), 4.20–4.14 (m, 2H), 3.29 (dd, *J* = 13.4, 3.2 Hz, 1H), 3.00–2.86 (m, 2H), 2.77 (dd, *J* = 13.4, 9.6 Hz, 1H), 1.72–1.66 (m, 2H), 1.38–1.23 (m, 16H), 0.88 (t, *J* = 6.9 Hz, 3H); ^13^C NMR (126 MHz, CDCl_3_) δ 173.56, 153.55, 135.44, 129.51, 129.01, 127.39, 66.23, 55.22, 38.00, 35.62, 31.99, 29.70, 29.57, 29.48, 29.42, 29.22, 24.37, 22.77, 14.19; one resonance was not observed due to coincidence of the chemical shifts. HRMS (ESI) *m/z* calcd. for C_22_H_33_NO_3_Na (M + Na)^+^: 382.2353, found 382.2354.

### 3.3. Synthesis of (S)-4-Benzyl-3-((S)-2-methyldodecanoyl)oxazolidin-2-one ((S,S)-***5***) (CAS 2771300-96-8)

NaHMDS (13 mL, 2.0 M in THF, 26.00 mmol) was added to a stirred solution of oxazolidinone amide **4** (6.25 g, 17.38 mmol) in dry THF (60 mL) at −78 °C over 15 min via syringe pump. The resulting mixture was stirred for 1 h at −78 °C, followed by slow addition of MeI (12.34 g, 86.93 mmol). The reaction mixture was maintained for 2 h at the same temperature, then quenched with saturated aqueous NH_4_Cl (20 mL). After the layers were separated, the aqueous phase was extracted with EtOAc (3 × 75 mL). The EtOAc extracts were combined with organic layer, washed with saturated aqueous NaCl (150 mL), dried over anhydrous Na_2_SO_4_, and filtered. The filtrate was concentrated under reduced pressure, and the residue was purified by silica gel column chromatography (EtOAc/petroleum ether 1:10) to afford (*S*)-4-benzyl-3-((*S*)-2-methyldodecanoyl) oxazolidin-2-one ((*S*,*S*)-**5**) (4.03 g, 62 % yield, dr > 99:1, based on ^13^C MNR spectra) as a white solid. [α]_D_^22^ = +56.5 (c = 2.04, CHCl_3_). ^1^H NMR (500 MHz, CDCl_3_) δ 7.34–7.21 (m, 5H), 4.68 (dd, *J* = 9.7, 7.2 Hz, 1H), 4.21–4.15 (m, 2H), 3.72–3.68 (m, 1H), 3.27 (dd, *J* = 13.4, 3.2 Hz, 1H), 2.77 (dd, *J* = 13.3, 9.6 Hz, 1H), 1.75–1.72 (m, 1H), 1.42–1.41 (m, 1H), 1.29–1.25 (m, 16H), 1.22 (d, *J* = 6.8 Hz, 3H), 0.88 (t, *J* = 6.9 Hz, 3H); ^13^C NMR (126 MHz, CDCl_3_) δ 177.49, 153.19, 135.49, 129.57, 129.05, 127.45, 66.12, 55.49, 38.04, 37.84, 33.57, 32.03, 29.79, 29.72, 29.64, 29.45, 27.40, 22.80, 17.48, 14.24; one resonance was not observed due to coincidence of the chemical shifts. HRMS (ESI) *m/z* calcd. for C_23_H_35_NO_3_Na (M + Na)^+^: 396.2509, found 396.2518. 

### 3.4. Synthesis of (S)-2-Methyldodecan-1-ol ((S)-***6***) (CAS 57289-26-6)

NaBH_4_ (2.02 g, 53.68 mmol) in water (8 mL) was added dropwise to a stirred solution of oxazolidinone amide **5** (4.01 g, 10.74 mmol) in THF (64 mL) at 0 °C over 30 min. After the reaction mixture was stirred for 3 h at room temperature, it was neutralized with aqueous HCl (1 M) until the pH was 6. The resulting mixture was then extracted with Et_2_O (3 × 30 mL). The combined organic layers were washed sequentially with saturated aqueous NaHCO_3_ (20 mL) and saturated aqueous NaCl (2 × 20 mL), dried over anhydrous Na_2_SO_4_, and filtered. The filtrate was concentrated under reduced pressure, and the residue was purified by silica gel column chromatography (EtOAc/petroleum ether 1:10) to afford (*S*)-2-methyldodecan-1-ol ((*S*)-**6**) (1.90 g, 88% yield, 98% ee, determined by chiral HPLC of its MBT derivative (*S*)-**8**). [α]_D_^22^ = −9.0 (c = 1.37, CHCl_3_), literature [21] [α]_D_^23^ = −8.4 (c = 1.00, CHCl_3_). ^1^H NMR (500 MHz, CDCl_3_) δ 3.51 (dd, *J* = 10.4, 5.8 Hz, 1H), 3.41 (dd, *J* = 10.4, 6.6 Hz, 1H), 1.64–1.59 (m, 2H), 1.26–1.16 (m, 17H), 0.91 (d, *J* = 6.7 Hz, 3H), 0.88 (d, *J* = 6.6 Hz, 3H); ^13^C NMR (126 MHz, CDCl_3_) δ 68.56, 35.91, 33.30, 32.06, 30.09, 29.81, 29.79, 29.78, 29.49, 27.13, 22.83, 16.72, 14.24. HRMS (ESI) *m/z* calcd. for C_13_H_28_OK (M + K)^+^: 239.1772, found 239.1786.

### 3.5. Synthesis of (S)-1-Bromo-2-methyldodecane ((S)-***7***) (CAS 1333499-05-0)

PPh_3_ (1.56 g, 5.97 mmol) and CBr_4_ (1.98 g, 5.97 mmol) were added to a stirred solution of alcohol **6** (1.14 g, 5.69 mmol) in DCM (30 mL) at 0 °C. The reaction mixture was warmed to room temperature and stirred for 6 h. The solvent was removed under reduced pressure and the residue was purified by silica gel column chromatography (*n*-hexane) to afford (*S*)-1-bromo-2-methyldodecane ((*S*)-**7**) (1.48 g, 99% yield) as a colorless oil. [α]_D_^22^ = −0.90 (c = 5.33, CHCl_3_). ^1^H NMR (500 MHz, CDCl_3_) δ 3.39 (dd, *J* = 9.8, 4.9 Hz, 1H), 3.32 (dd, *J* = 9.8, 6.3 Hz, 1H), 1.80–1.76 (m, 1H), 1.31–1.18 (m, 18H), 1.01 (d, *J* = 6.6 Hz, 3H), 0.88 (t, *J* = 6.9 Hz, 3H); ^13^C NMR (126 MHz, CDCl_3_) δ 41.75, 35.37, 35.04, 32.07, 29.86, 29.78, 29.74, 29.50, 27.04, 22.84, 18.95, 14.26; one resonance was not observed due to coincidence of the chemical shifts. HRMS (ESI) *m/z* calcd. for C_13_H_27_Br (M)^+^: 262.1291, found 262.1279.

### 3.6. Synthesis of (R)-4-Benzyl-3-dodecanoyloxazolidin-2-one ((R)-***4***) (CAS 185803-85-4)

According to the same manner of (*S*)-**4**, dodecanoic acid (**2**) (5.00 g, 24.97 mmol), oxalyl chloride (4.75 g, 37.43 mmol), and (*R*)-4-benzyloxazolidin-2-one ((*R*)-**3**) (2.95 g, 16.64 mmol, >99% ee) afforded (*R*)-4-benzyl-3-dodecanoyloxazolidin-2-one ((*R*)-**4**) as a colorless oil (5.67 g, 95% yield). [α]_D_^22^ = −31.0 (c = 2.18, CHCl_3_). ^1^H NMR (500 MHz, CDCl_3_) δ 7.35–7.17 (m, 5H), 4.70–4.67 (m, 1H), 4.21–4.15 (m, 2H), 3.30 (dd, *J* = 13.4, 3.3 Hz, 1H), 3.00–2.86 (m, 2H), 2.76 (dd, *J* = 13.4, 9.6 Hz, 1H), 1.73–1.66 (m, 2H), 1.30–1.26 (m, 16H), 0.87 (t, *J* = 7.1 Hz, 3H); ^13^C NMR (126 MHz, CDCl_3_) δ 173.62, 153.61, 135.48, 129.57, 129.09, 127.47, 66.28, 55.29, 38.06, 35.68, 32.04, 29.76, 29.72, 29.62, 29.54, 29.47, 29.27, 24.40, 22.82, 14.25. HRMS (ESI) *m/z* calcd. for C_22_H_33_NO_3_Na (M + Na)^+^: 382.2353, found 382.2364.

### 3.7. Synthesis of (R)-4-Benzyl-3-((R)-2-methyldodecanoyl)oxazolidin-2-one ((R,R)-***5***) (CAS 185803-86-5)

According to the same manner of (*S*,*S*)-**5**, oxazolidinone amide (*R*)-**4** (6.25 g, 17.4 mmol) and MeI (12.34 g, 86.93 mmol) afforded (*R*)-4-benzyl-3-((*R*)-2-methyldodecanoyl)oxazolidin-2-one ((*R,R*)-**5**) (4.85 g, 75% yield, dr > 99:1, based on ^13^C MNR spectra) as a white solid. [α]22D = −71.3 (c = 2.31, CHCl_3_). ^1^H NMR (500 MHz, CDCl_3_) δ 7.34–7.21 (m, 5H), 4.67 (ddt, *J* = 10.3, 6.8, 3.1 Hz, 1H), 4.21–4.15 (m, 2H), 3.72–3.67 (m, 1H), 3.26 (dd, *J* = 13.3, 3.3 Hz, 1H), 2.77 (dd, *J* = 13.3, 9.5 Hz, 1H), 1.76–1.70 (m, 1H), 1.44–1.38 (m, 1H), 1.28–1.23 (m, 16H), 1.22 (d, *J* = 6.9 Hz, 3H), 0.88 (t, *J* = 6.9 Hz, 3H); ^13^C NMR (126 MHz, CDCl_3_) δ 177.49, 153.18, 135.49, 129.57, 129.03, 127.43, 66.10, 55.46, 38.01, 37.82, 33.55, 32.01, 29.77, 29.71, 29.62, 29.43, 27.37, 22.79, 17.46, 14.21; one resonance was not observed due to coincidence of the chemical shifts. HRMS (ESI) *m*/*z* calcd. for C_23_H_35_NO_3_Na (M + Na)^+^: 396.2509, found 396.2516.

### 3.8. Synthesis of (R)-2-Methyldodecan-1-ol ((R)-***6***) (CAS 109034-03-9)

According to the same manner of (*S*)-**6**, oxazolidinone amide (*R,R*)-**5** (3.50 g, 9.37 mmol) and NaBH_4_ (1.77 g, 46.85 mmol) afforded (*R*)-2-methyldodecan-1-ol ((*R*)-**6**) (1.60 g, 85% yield, 99% ee, determined by chiral HPLC of its MBT derivative (*R*)-**8**) as a colorless oil. [α]_D_^22^ = +10.8 (c = 1.52, CHCl_3_), literature [31] [α]_D_^22^ = +9.1 (c = 0.80, CHCl_3_). ^1^H NMR (500 MHz, CDCl_3_) δ 3.50 (dd, *J* = 10.5, 5.8 Hz, 1H), 3.42 (dd, *J* = 10.5, 6.5 Hz, 1H), 1.63–1.35 (m, 2H), 1.27–1.13 (m, 17H), 0.91 (d, *J* = 6.8 Hz, 3H), 0.88 (t, *J* = 6.9 Hz, 3H); ^13^C NMR (126 MHz, CDCl_3_) δ 68.55, 35.90, 33.30, 32.06, 30.09, 29.81, 29.78, 29.48, 27.12, 22.82, 16.71, 14.24; one resonance was not observed due to coincidence of the chemical shifts. HRMS (ESI) *m/z* calcd. for C_13_H_28_OK (M + K)^+^: 239.1772, found 239.1785.

### 3.9. Synthesis of (R)-1-Bromo-2-methyldodecane ((R)-***7***) (CAS 1643618-98-7)

According to the same manner of (*S*)-**7**, alcohol (*R*)-**6** (1.50 g, 7.49 mmol), PPh_3_ (2.06 g, 7.86 mmol), and CBr_4_ (2.61 g, 7.86 mmol) afforded (*R*)-1-bromo-2-methyldodecane ((*R*)-**7**) (1.87 g, 95% yield) as a colorless oil. [α]_D_^22^ = +0.29 (c = 3.99, CHCl_3_). ^1^H NMR (500 MHz, CDCl_3_) δ 3.40 (dd, *J* = 9.8, 4.9 Hz, 1H), 3.32 (dd, *J* = 9.8, 6.3 Hz, 1H), 1.81–1.75 (m, 1H), 1.45–1.42 (m, 1H), 1.33–1.19 (m, 18H), 1.01 (d, *J* = 6.6 Hz, 3H), 0.88 (t, *J* = 6.9 Hz, 3H); ^13^C NMR (126 MHz, CDCl_3_) δ 41.77, 35.36, 35.03, 32.06, 29.86, 29.77, 29.73, 29.48, 27.03, 22.83, 18.95, 14.25; one resonance was not observed due to coincidence of the chemical shifts. HRMS (ESI) *m*/*z* calcd. for C_13_H_28_Br (M + H)^+^: 263.1369, found 263.1369.

### 3.10. Synthesis of 2-((2-Methyldodecyl)thio)benzo[d]thiazole (rac-***8***) (New Compound)

Ph_3_P (0.38 g, 1.44 mmol) was added to a stirred solution of alcohol *rac*-**6** (0.24 g, 1.20 mmol) in THF (6 mL) at 0 °C. Benzo[*d*]thiazole-2(*3H*)-thione (MBT) (0.24 g, 1.44 mmol) and DIAD (0.29 g, 1.44 mmol) were added sequentially. The reaction mixture was warmed to room temperature and stirred for 4 h, followed by removal of solvent under reduced pressure. The residue was purified by silica gel column chromatography (hexanes: ethyl acetate = 50:1) to afford 2-((2-methyldodecyl)thio)benzo[*d*]thiazole (*rac*-**8**) (0.31 g, 75% yield) as a yellow oil. ^1^H NMR (500 MHz, CDCl_3_) δ 7.77 (dd, *J* = 8.1, 1.0 Hz, 1H), 7.66 (dd, *J* = 8.0, 1.2 Hz, 1H), 7.32 (td, *J* = 8.2, 7.7, 1.2 Hz, 1H), 7.20 (td, *J* = 7.6, 1.1 Hz, 1H), 3.33 (dd, *J* = 12.7, 5.6 Hz, 1H), 3.11 (dd, *J* = 12.7, 7.5 Hz, 1H), 1.87–1.83 (m, 1H), 1.45–1.41 (m, 1H), 1.21–1.18 (m, 17H), 1.06 (d, *J* = 6.7 Hz, 3H), 0.88 (t, *J* = 6.9 Hz, 3H); ^13^C NMR (126 MHz, CDCl_3_) δ 167.89, 153.51, 135.31, 126.10, 124.20, 121.57, 121.02, 36.24, 33.37, 32.05, 29.89, 29.79,29.78, 29.74, 29.48, 27.03, 22.82, 19.51, 14.26; one resonance was not observed due to coincidence of the chemical shifts. HRMS (ESI) *m*/*z* calcd for C_20_H_32_NS_2_ (M + H)^+^: 350.1971, found 350.1960.

### 3.11. Synthesis of (S)-2-((2-Methyldodecyl)thio)benzo[d]thiazole ((S)-***8***) (New Compound)

According to the same manner of *rac***-8**, alcohol (*S*)-**6** (0.080 g, 0.40 mmol) and benzo[*d*]thiazole-2(*3H*)-thione (0.080 g, 0.48 mmol) afforded (*S*)-2-((2-methyldodecyl)thio) benzo[*d*]thiazole ((*S*)-**8** (0.11 g, 79% yield, 98% ee) as a yellow oil. The ee was determined by chiral HPLC (Daicel Chiralcel OD-H column, 1% isopropanol in *n*-hexane, 0.7 mL/min, 254 nm, major t_r_ = 8.53 min (*S*), minor t_r_ = 9.40 min (*R*)). [α]_D_^22^ = +7.7 (c = 1.46, CHCl_3_). ^1^H NMR (500 MHz, CDCl_3_) δ 7.78–7.76 (m, 1H), 7.65 (dd, *J* = 8.0, 1.2 Hz, 1H), 7.33–7.29 (m, 1H), 7.21–7.17 (m, 1H), 3.33 (dd, *J* = 12.7, 5.7 Hz, 1H), 3.11 (dd, *J* = 12.7, 7.5 Hz, 1H), 1.87–1.83 (m, 1H), 1.45–1.40 (m, 1H), 1.21–1.18 (m, 17H), 0.98 (d, *J* = 6.7 Hz, 3H), 0.80 (t, *J* = 6.9 Hz, 3H); ^13^C NMR (126 MHz, CDCl_3_) δ 167.89, 153.49, 135.29, 126.09, 124.19, 121.55, 121.01, 40.84, 36.23, 33.35, 32.05, 29.89, 29.78, 29.75, 29.49, 27.03, 22.83, 19.51, 14.26; one resonance was not observed due to coincidence of the chemical shifts. HRMS (ESI) *m*/*z* calcd for C_20_H_32_NS_2_ (M + H)^+^: 350.1971, found 350.1973. 

### 3.12. Synthesis of (R)-2-((2-Methyldodecyl)thio)benzo[d]thiazole ((R)-***8***) (New Compound)

According to the same manner of *rac*-**8**, alcohol (*R*)-**6** (0.080 g, 0.40 mmol) and benzo[*d*]thiazole-2(*3H*)-thione (0.080 g, 0.48 mmol) afforded (*R*)-2-((2-methyldodecyl)thio)benzo[*d*]thiazole ((*R*)-**8**) (0.11 g, 79% yield, 99% ee) as a yellow oil. The ee was determined by chiral HPLC (Daicel Chiralcel OD-H column, 1% isopropanol in *n*-hexane, 0.7 mL/min, 254 nm, minor t_r_ = 8.78 min (*S*), major t_r_ = 9.44 min (*R*)). [α]_D_^22^ = -8.0 (c = 1.84, CHCl_3_). ^1^H NMR (500 MHz, CDCl_3_) δ 7.79–7.77 (m, 1H), 7.65 (dd, *J* = 8.0, 1.2 Hz, 1H), 7.34–7.31 (m, 1H), 7.21–7.19 (m, 1H), 3.33 (dd, *J* = 12.7, 5.7 Hz, 1H), 3.11 (dd, *J* = 12.7, 7.5 Hz, 1H), 1.86–1.84 (m, 1H), 1.46–1.40 (m, 1H), 1.18–1.15 (m, 17H), 0.98 (d, *J* = 6.7 Hz, 3H), 0.81 (t, *J* = 6.9 Hz, 3H); ^13^C NMR (126 MHz, CDCl_3_) δ 167.92, 153.51, 135.30, 126.11, 124.20, 121.56, 121.02, 40.86, 36.24, 33.37, 32.06, 29.89, 29.78, 29.75, 29.49, 27.03, 22.83, 19.52, 14.26; one resonance was not observed due to coincidence of the chemical shifts. HRMS (ESI) *m*/*z* calcd for C_20_H_32_NS_2_ (M + H)^+^: 350.1971, found 350.1972. 

### 3.13. Synthesis of (S)-11-Methylheptacos-9-ene ((S)-***10***) (New Compound)

Ph_3_P (3.31 g, 12.62 mmol) was added to a stirred solution of hydrocarbon bromide (*S*)-**7** (2.20 g, 8.35 mmol) in CH_3_CN (60 mL) at room temperature. After the reaction solution was heated to 85 °C and stirred for 48 h, it was cooled to room temperature and concentrated under reduced pressure. The residue was purified by silica gel column chromatography (DCM/MeOH 10:1) to afford the corresponding phosphonium salt (2.23 g, 51% yield) as a colorless oil.

*n*-BuLi (0.25 mL, 2.4 M in *n*-hexane, 0.60 mmol) was added dropwise to a stirred solution of phosphonium salt (0.24 g, 0.45 mmol) in dry THF (5 mL) at room temperature via syringe. After the reaction mixture was maintained for 2 h at the same temperature, it was cooled to −35 °C. *n*-Pentadecanal (**9**) (0.068 g, 0.30 mmol) in dry THF (3 mL) was then added. The reaction mixture was stirred for 5 h at −35 °C and quenched with saturated aqueous NH_4_Cl (5 mL). After the layers were separated, the aqueous phase was extracted with Et_2_O (3 × 5 mL). The ether extracts were combined with the organic layer, washed with saturated aqueous NaCl (20 mL), dried over anhydrous Na_2_SO_4_, and filtered. The filtrate was concentrated under reduced pressure, and the residue was purified by silica gel column chromatography (petroleum ether) to give the *Z*/*E* mixtures of (*S*)-11-methylheptacos-9-ene ((*S*)-**10**) (0.062 g, 53% yield, *Z*:*E* = 5.1:1, determined by ^13^C NMR spectra) as a colorless liquid. [α]_D_^22^ = −1.09 (c = 1.47, CHCl_3_). ^1^H NMR (500 MHz, CDCl_3_) δ 5.31–5.26 (m, 1H), 5.12–5.08 (m, 1H), 2.41–2.40 (m, 1H), 2.03–1.95 (m, 2H), 1.29–1.24 (m, 42H), 0.91 (d, *J* = 6.7 Hz, 3H), 0.88 (t, *J* = 6.9 Hz, 6H); ^13^C NMR (126 MHz, CDCl_3_) δ 135.63, 135.58, 127.59, 127.50, 76.42, 76.16, 75.91, 36.77, 31.10, 30.78, 29.12, 29.01, 28.87, 28.84, 28.75, 28.54, 26.70, 26.65, 21.86, 20.60, 13.28. EIMS (*m*/*z*(%)): 392.5(8, M^+^), 266.3(9), 167.1(19), 139.1(12), 83.1(78), 71.1(52), 57.1(100), 43.1(59). 

### 3.14. Synthesis of (S)-11-Methylheptacosane ((S)-***1***) (CAS 1370709-05-9)

Pd (0.010 g, 10% on carbon) was placed in a 25-mL Schlenk tube, and hydrogen was charged at room temperature. Olefine (*S*)-**10** (0.033 g, 0.084 mmol) in EtOH (5 mL) was then added dropwise. The reaction mixture was maintained for 12 h, during which time hydrogen was bubbled into the Schlenk tube from a hydrogen balloon. After the reaction mixture was filtered, the filter was washed with *n*-hexane (10 mL). The filtrate and washing were combined and concentrated under reduced pressure. The residue was purified by silica gel column chromatography (*n*-hexane) to obtain (*S*)-11-methylheptacosane ((*S*)-**1**) (0.018 g, 54% yield) as a colorless oil. [α]_D_^22^ = −3.33 (c = 0.60, CHCl_3_), literature [13] [α]_D_^20^ = −0.06 (c = 3.33, hexanes). ^1^H NMR (500 MHz, CDCl_3_) δ 1.38–1.35 (m, 1H), 1.31–1.26 (m, 48H), 0.88 (t, *J* = 6.8 Hz, 6H), 0.83 (d, *J* = 6.5 Hz, 3H); ^13^C NMR (126 MHz, CDCl_3_) δ 37.25, 32.90, 32.08, 30.18, 29.89, 29.85, 29.81, 29.51, 27.24, 22.84, 19.88, 14.27; sixteen resonances were not observed due to coincidence of the chemical shifts. EIMS (*m*/*z*(%)): 379.5(3, (M−Me)^+^), 252.3(14), 168.2(34), 99.1(26), 85.1(65), 71.1(81), 57.1(100), 43.1(54). 

### 3.15. Synthesis of (R)-11-Methylheptacos-9-ene ((R)-***10***) (New Compound)

According to the same manner of (*S*)-**10**, hydrocarbon bromide (*R*)-**7** (2.86 g, 10.86 mmol) and Ph_3_P (4.30 g, 16.39 mmol) provided the corresponding phosphonium salt (2.90 g, 51% yield) as a colorless oil, followed by the reaction with *n*-pentadecanal (**9**) (0.15 g, 0.66 mmol) affording the *Z*/*E* mixtures of (*R*)-11-methylheptacos-9-ene ((*R*)-**10**) (0.020 g, 77% yield, *Z*:*E* = 2.2:1, determined by ^13^C NMR spectra) as a colorless liquid. [α]_D_^22^ = +7.09 (c = 0.73, CHCl_3_). ^1^H NMR (500 MHz, CDCl_3_) δ 5.34–5.27 (m, 1H), 5.26–5.07 (m, 1H), 2.04–1.94 (m, 2H), 1.33–1.25 (m, 43H), 0.94–0.90 (m, 3H), 0.88 (t, *J* = 6.8 Hz, 6H); ^13^C NMR (126 MHz, CDCl_3_) δ 136.64, 136.59, 128.60, 128.51, 37.77, 37.41, 36.87, 32.74, 32.10, 31.77, 30.11, 30.00, 29.95, 29.87, 29.83, 29.74, 29.69, 29.53, 29.29, 27.69, 27.65, 27.51, 22.86, 21.59, 21.12, 14.27. EIMS (*m*/*z*(%)): 392.4(7, M^+^), 266.3(10), 167.2(19), 139.1(12), 83.1(80), 71.1(51), 57.1(100), 43.1(58). 

### 3.16. Synthesis of (R)-11-Methylheptacosane ((R)-***1***) (CAS 1370709-06-0)

According to the same manner of (*S*)-**1**, Pd (0.023g, 10% on carbon) and olefine (*R*)-**10** (0.077 g, 0.20 mmol) afforded (*R*)-11-methylheptacosane ((*R*)-**1**) (0.052 g, 66% yield) as a colorless oil. [α]_D_^22^ = +3.31 (c = 1.43, CHCl_3_), literature [13] [α]_D_^20^ = +0.09 (c = 4.68, hexanes). ^1^H NMR (500 MHz, CDCl_3_) δ 1.37–1.35 (m, 1H), 1.31–1.26 (m, 48H), 0.88 (t, *J* = 6.8 Hz, 6H), 0.83 (d, *J* = 6.5 Hz, 3H); ^13^C NMR (126 MHz, CDCl_3_) δ 37.25, 32.90, 32.08, 30.19, 29.89, 29.85, 29.82, 29.52, 27.24, 22.85, 19.88, 14.27. Sixteen resonances were not observed due to coincidence of the chemical shifts. EIMS (*m*/*z*(%)): 379.5(2, (M−Me)^+^), 252.3(14), 168.2(32), 99.1(25), 85.1(65), 71.1(79), 57.1(100), 43.1(51).

## 4. Conclusions

In summary, we have developed a novel and efficient synthesis of (*R*)-11-methylheptacosane, the contact sex pheromone of *Tetropium fuscum*, and its enantiomer. The central element to our strategy involved Evans’ chiral auxiliary to construct the stereocenter, and Wittig coupling to connect aldehyde with chiral phosphonium salt. The synthetic pheromones would be helpful for the development of the pest control.

## Data Availability

The data presented in this article are available in the Supplementary Materials.

## Supplementary Materials

^1^H NMR and ^13^C NMR spectra for all the synthetic compounds and chiral HPLC chromatography of the MBT derivatives **8** can be downloaded at: https://www.mdpi.com/article/10.3390/molecules27206897/s1.
