# Supplementary material for: Asymmetric Synthesis of Contact Sex Pheromone of Tetropium fuscum and Its Enantiomer
Source: Molecules. 2022 Oct 14;27(20):6897. doi: 10.3390/molecules27206897 (PMC9611917; doi:10.3390/molecules27206897)
Supplement: Supplementary file 1 [file molecules-27-06897-s001.zip › molecules-1964432-supplementary.pdf]

SUPPORTING INFORMATION

Asymmetric Synthesis of Contact Sex Pheromone of *Tetropium fuscum* and Its Enantiomer

Xueyang Wang [1], Jianan Wang [1], Fengbo Ma [2], Qinghua Bian [1], Min Wang [1] and Jiangchun Zhong [1,*]

[1] Department of Applied Chemistry, China Agricultural University, 2 West Yuanmingyuan Road, Beijing 100193, China
[2] College of Enviromental Sciences, Sichuan Agricultural University, 211 Huimin Road, Chengdu 611130, China
* Correspondence: zhong@cau.edu.cn; Tel.: +86-010-6273-1356

Table of Contents

1. ^{1}H, ^{13}C NMR Spectra of the Products……………………………………………………….............S2

2. HPLC Chromatography of the Compounds………………………………………..………..S17

1. ¹H, ¹³C NMR Spectra of the Products

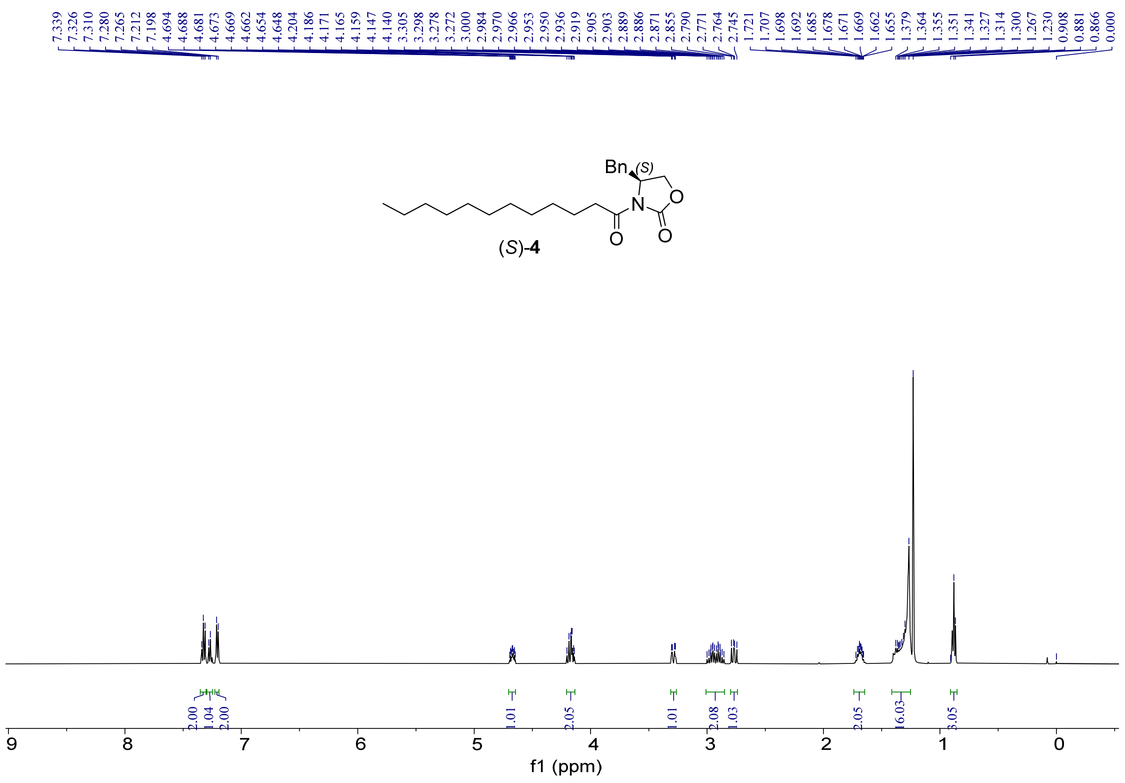

Figure S1. ¹H NMR Spectrum of (*S*)-4-benzyl-3-dodecanoyloxazolidin-2-one ((*S*)-**4**) (500 MHz, $CDCl_3$)

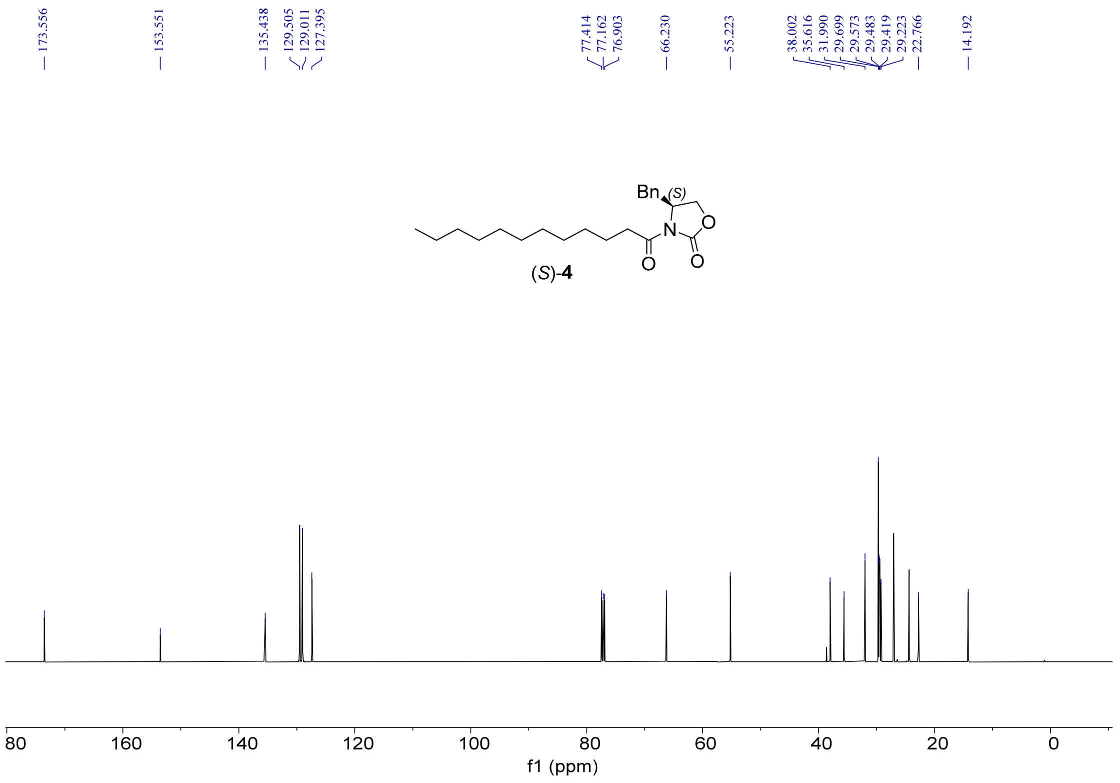

Figure S2. ¹³C NMR Spectrum of (*S*)-4-benzyl-3-dodecanoyloxazolidin-2-one ((*S*)-**4**) (126 MHz, $CDCl_3$)

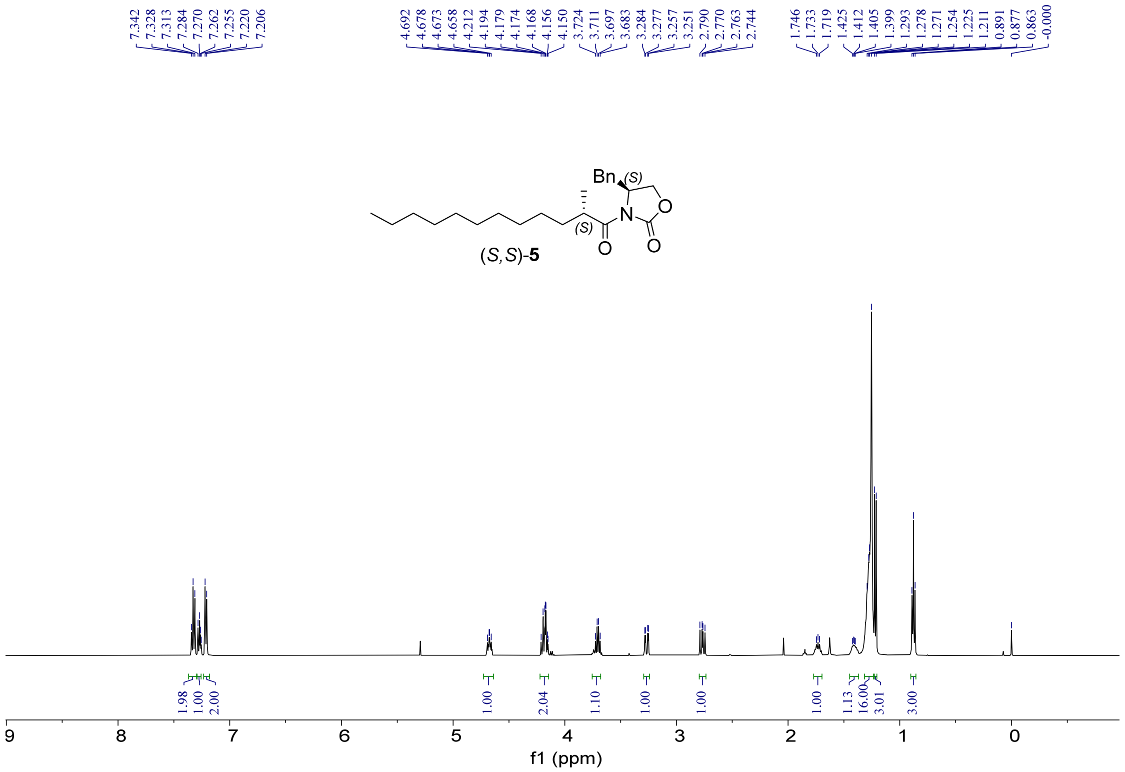

Figure S3. ^{1}H NMR Spectrum of (*S*)-4-benzyl-3-((S)-2-methyldodecanoyl)oxazolidin-2-one ((*S*,*S*)-**5**) (500 MHz, $CDCl_3$)

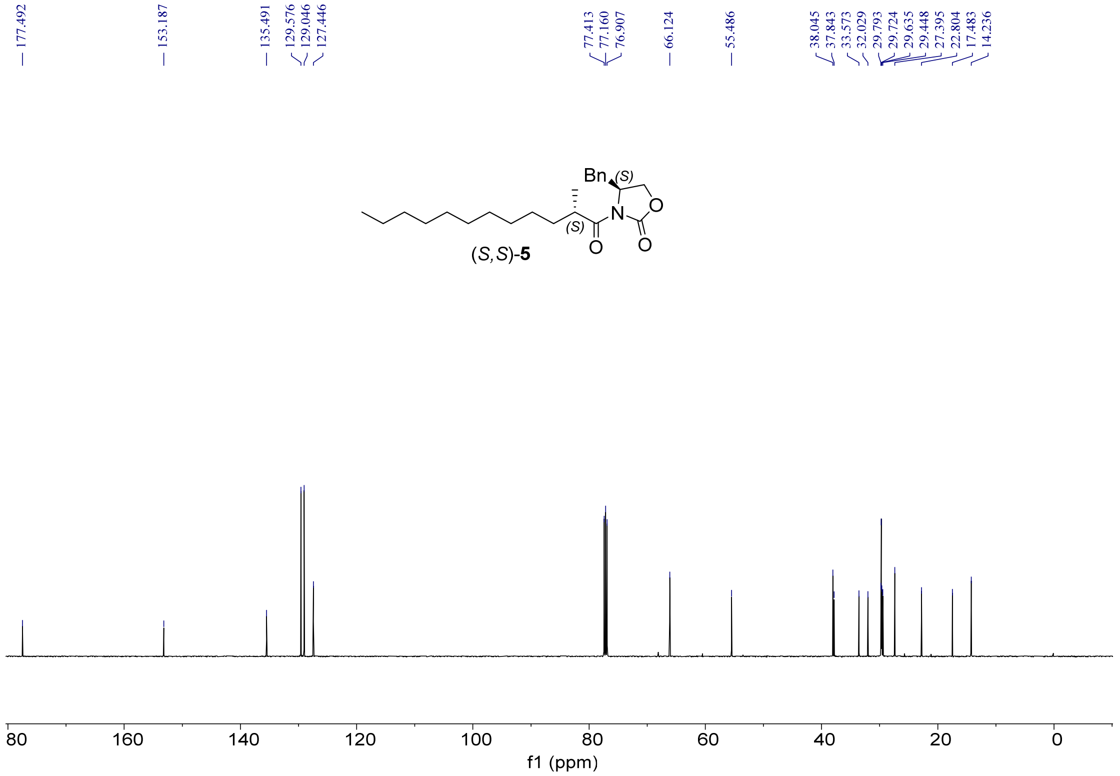

Figure S4. ^{13}C NMR Spectrum of (*S*)-4-benzyl-3-((S)-2-methyldodecanoyl)oxazolidin-2-one ((*S*,*S*)-**5**) (126 MHz, $CDCl_3$)

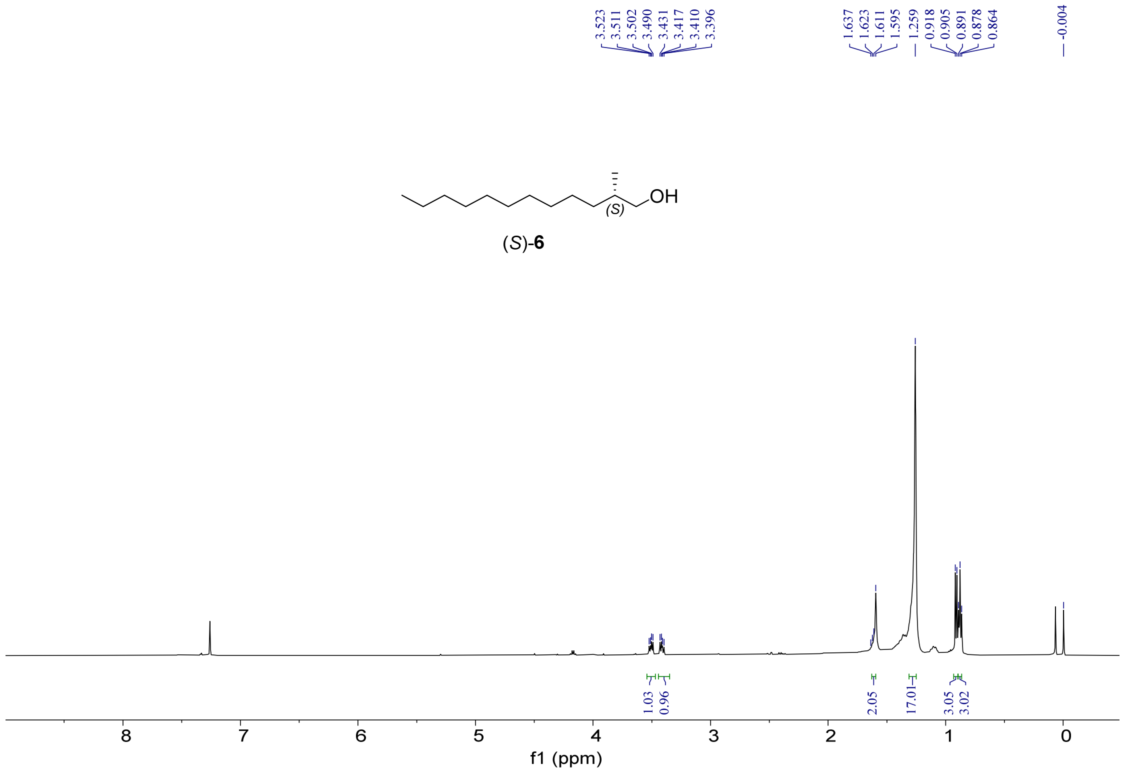

Figure S5. ^{1}H NMR Spectrum of(*S*)-2-methyldodecan-1-ol ((*S*)-**6**) (500 MHz, $CDCl_3$)

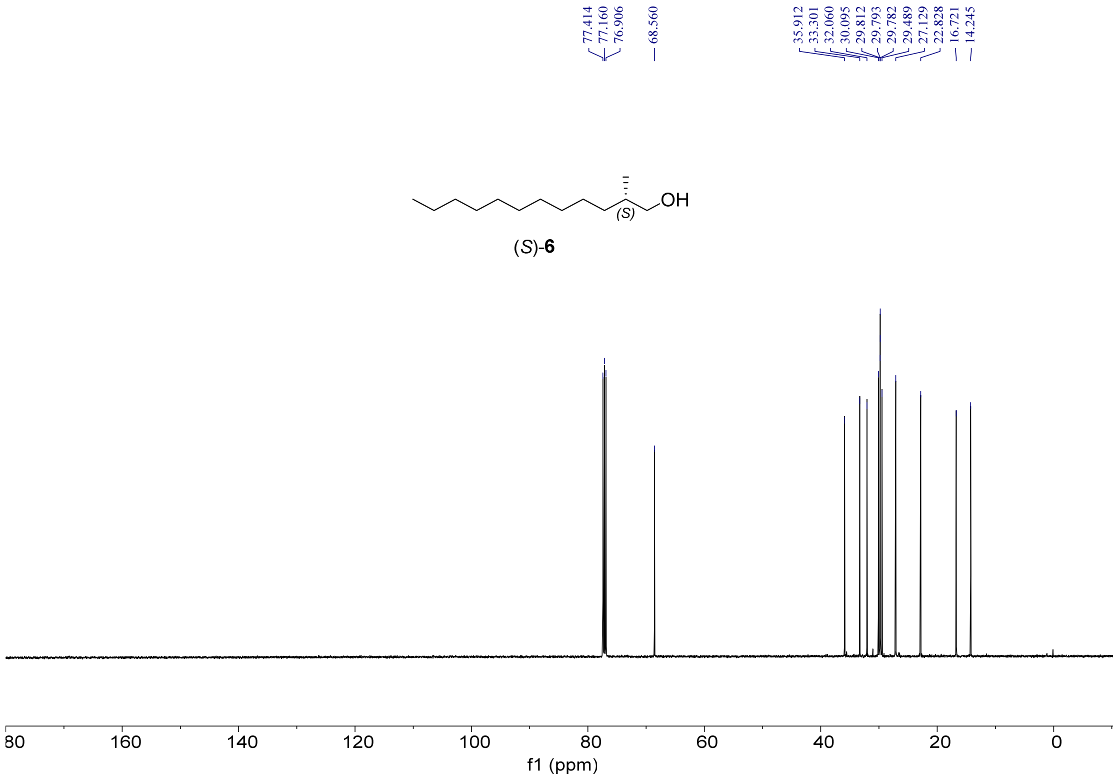

Figure S6. ^{13}C NMR Spectrum of (*S*)-2-methyldodecan-1-ol ((*S*)-**6**) (126 MHz, $CDCl_3$)

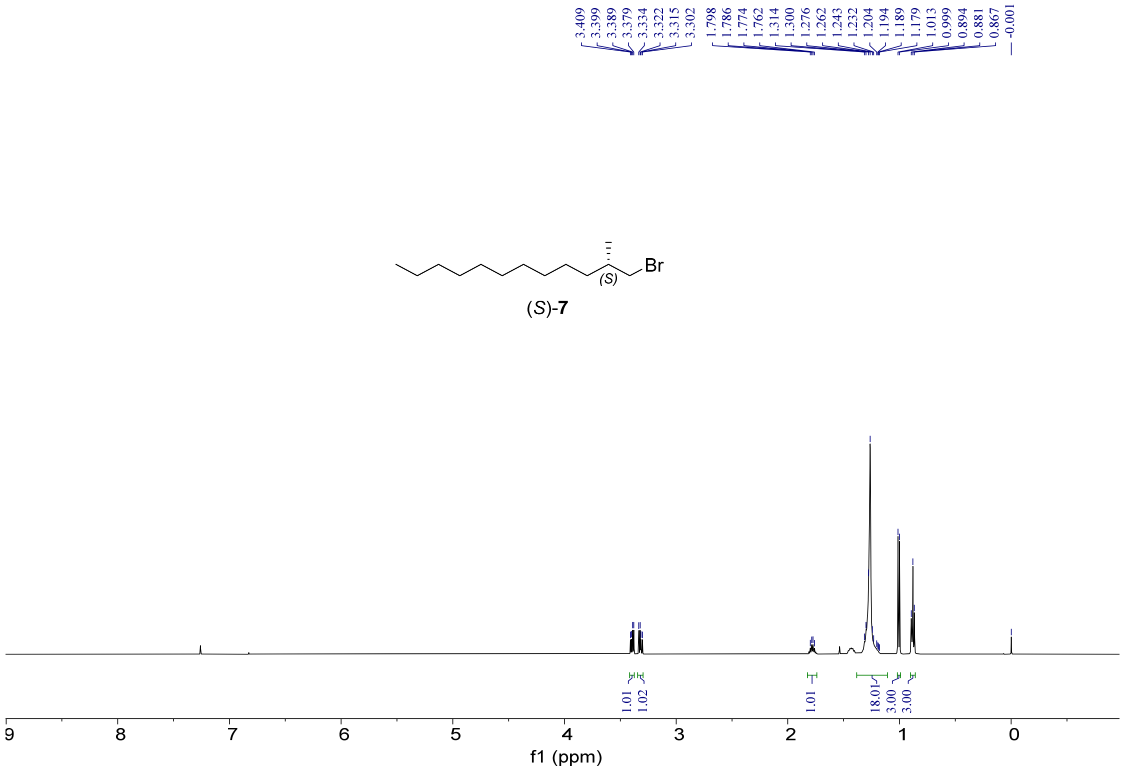

Figure S7. ^{1}H NMR Spectrum of (*S*)-1-bromo-2-methyldodecane ((*S*)-**7**) (500 MHz, $CDCl_3$)

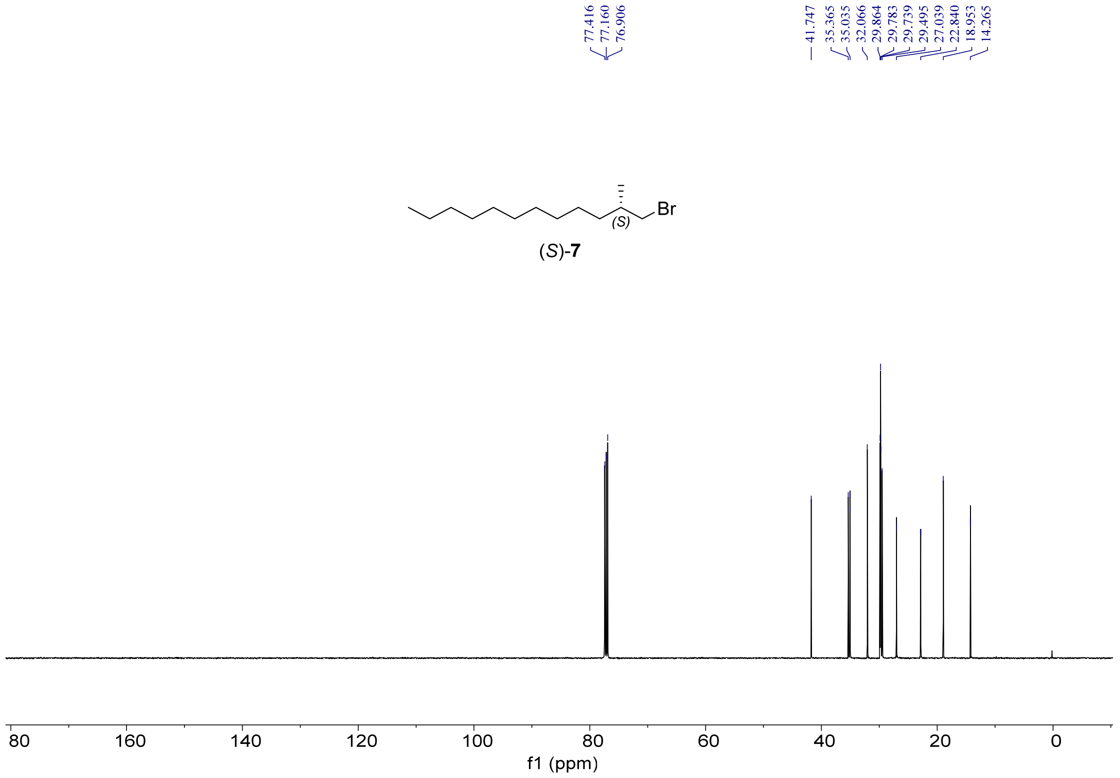

Figure S8. ^{13}C NMR Spectrum of (*S*)-1-bromo-2-methyldodecane ((*S*)-**7**) (126 MHz, $CDCl_3$)

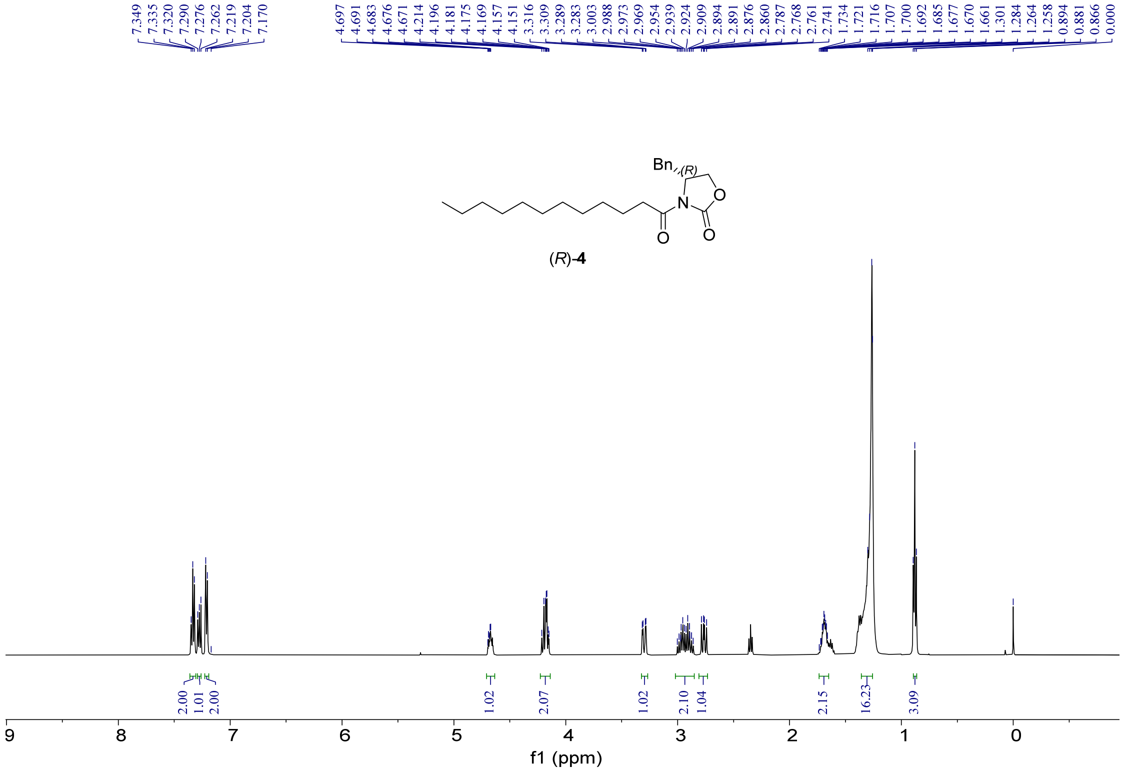

Figure S9. ^{1}H NMR Spectrum of (*R*)-4-benzyl-3-dodecanoyloxazolidin-2-one ((*R*)-**4**) (500 MHz, $CDCl_3$)

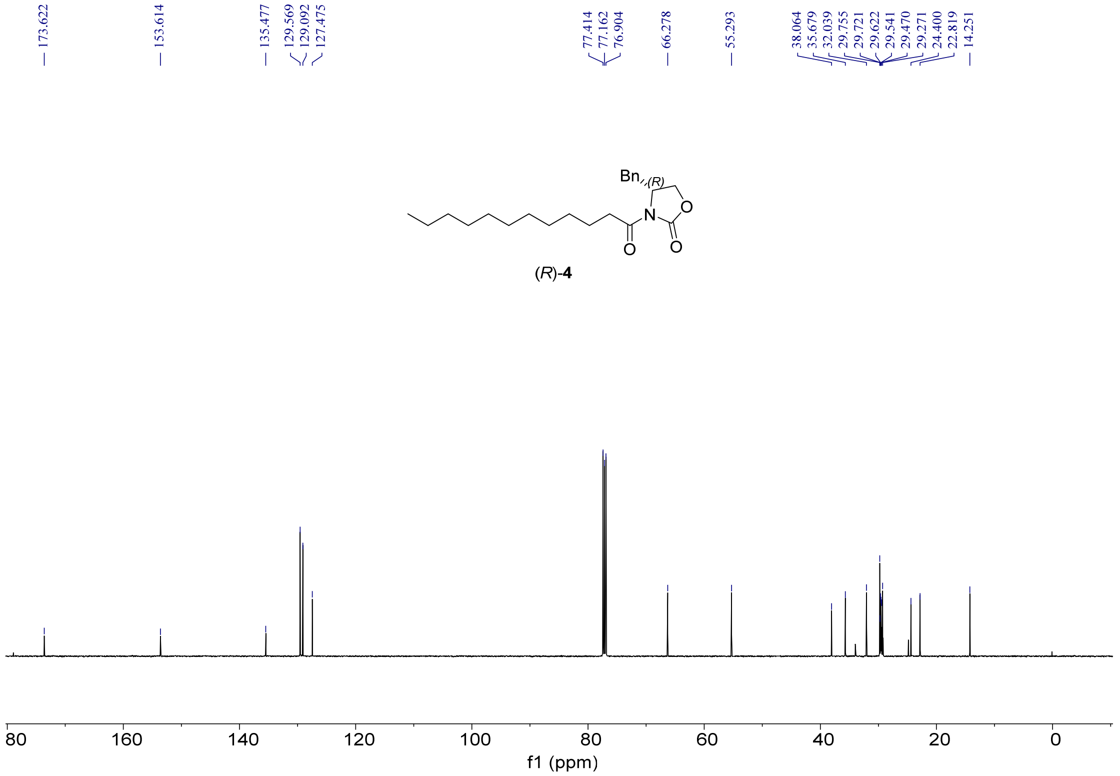

Figure S10. ^{13}C NMR Spectrum of (*R*)-4-benzyl-3-dodecanoyloxazolidin-2-one ((*R*)-**4**) (126 MHz, $CDCl_3$)

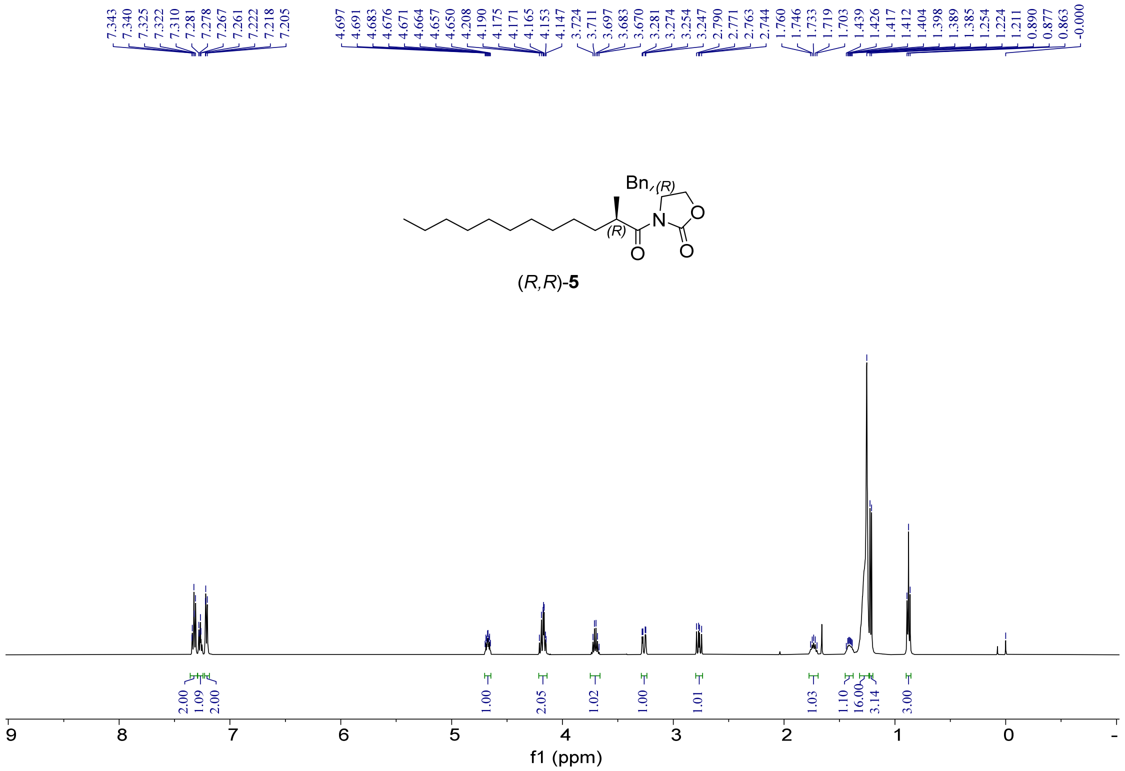

Figure S11. ^{1}H NMR Spectrum of (*R*)-4-benzyl-3-((*R*)-2-methyldodecanoyl)oxazolidin-2-one((*R*,*R*)-**5**) (500 MHz, $CDCl_3$)

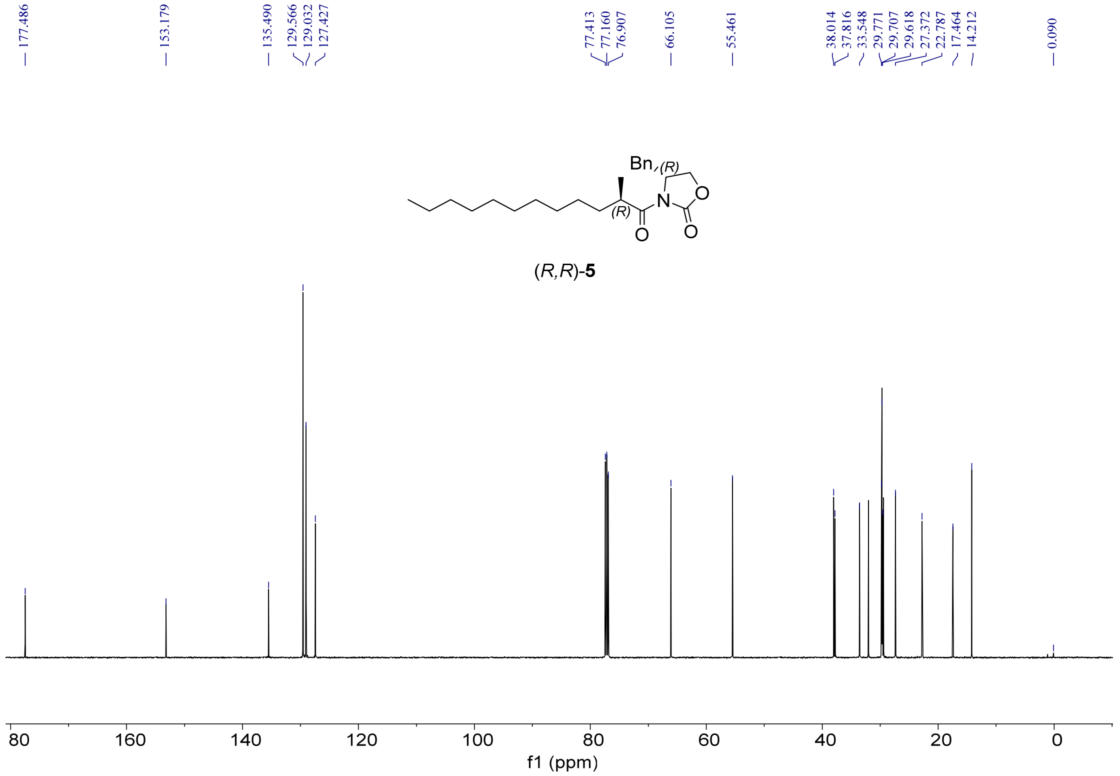

Figure S12. ^{13}C NMR Spectrum of (*R*)-4-benzyl-3-((*R*)-2-methyldodecanoyl)oxazolidin-2-one((*R*,*R*)-**5**) (126 MHz, $CDCl_3$)

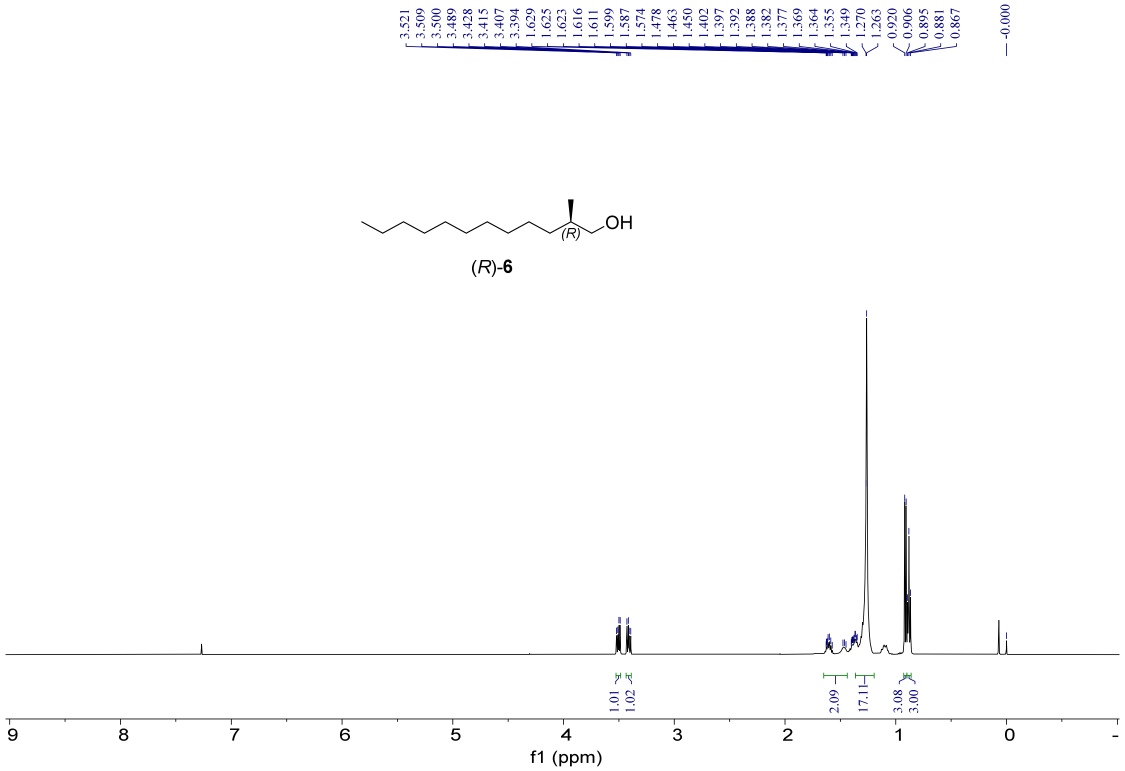

Figure S13. ^{1}H NMR Spectrum of (*R*)-2-methyldodecan-1-ol ((*R*)-**6**) (500 MHz, $CDCl_3$)

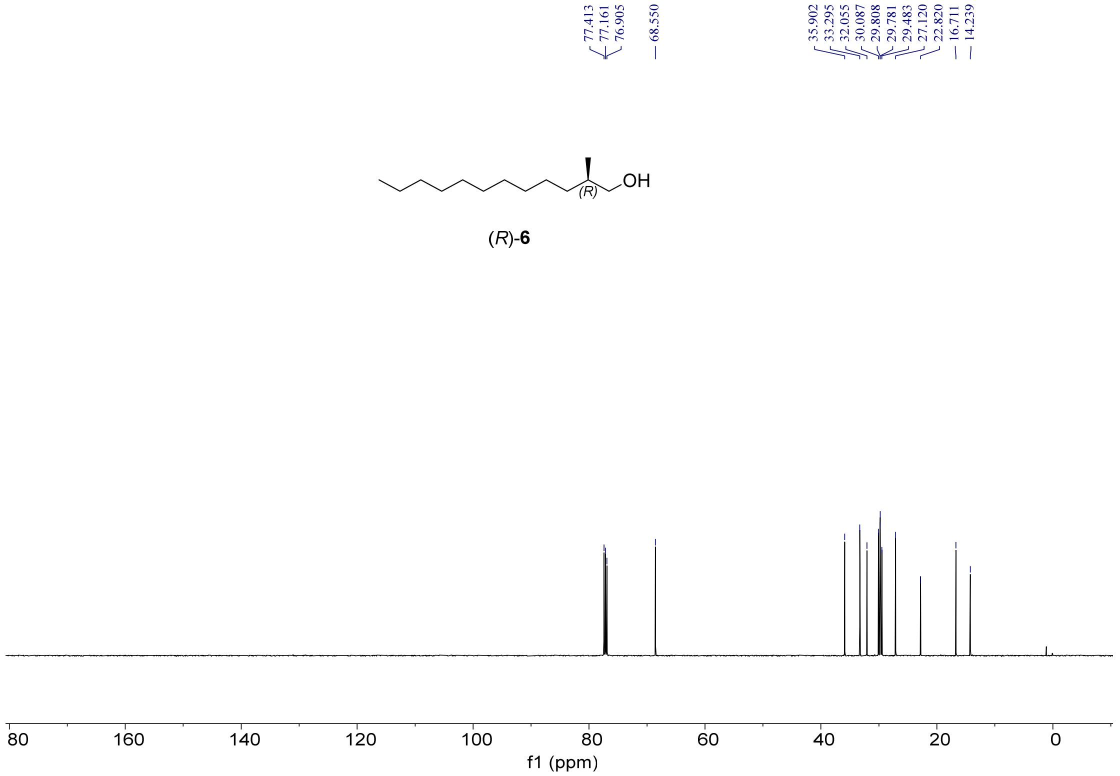

Figure S14. ^{13}C NMR Spectrum of (*R*)-2-methyldodecan-1-ol ((*R*)-**6**) (126 MHz, $CDCl_3$)

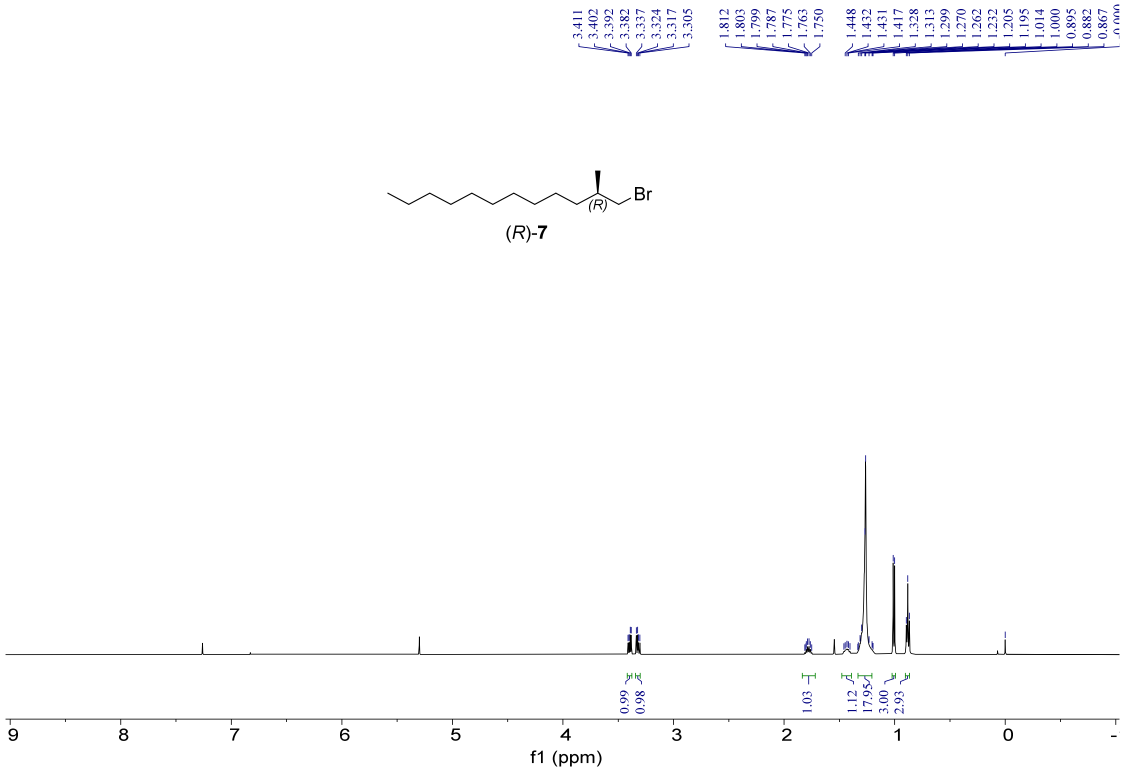

Figure S15. ^{1}H NMR Spectrum of (*R*)-1-bromo-2-methyldodecane ((*R*)-**7**) (500 MHz, $CDCl_3$)

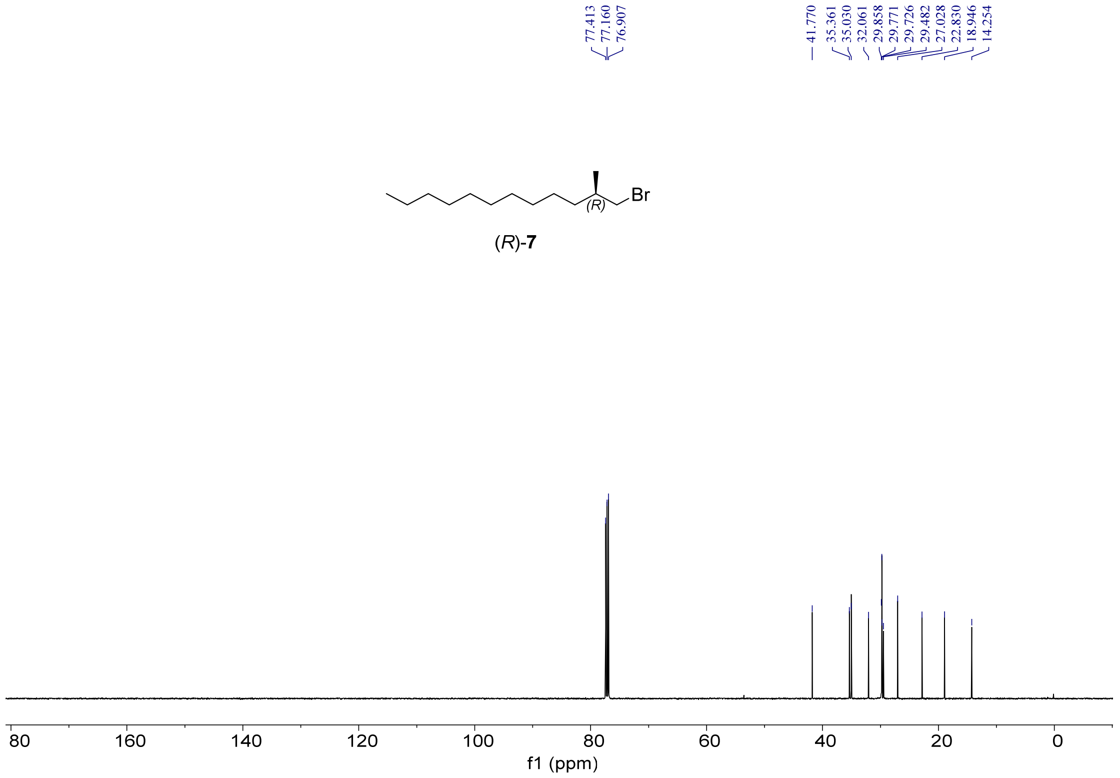

Figure S16. ^{13}C NMR Spectrum of (*R*)-1-bromo-2-methyldodecane ((*R*)-**7**) (126 MHz, $CDCl_3$)

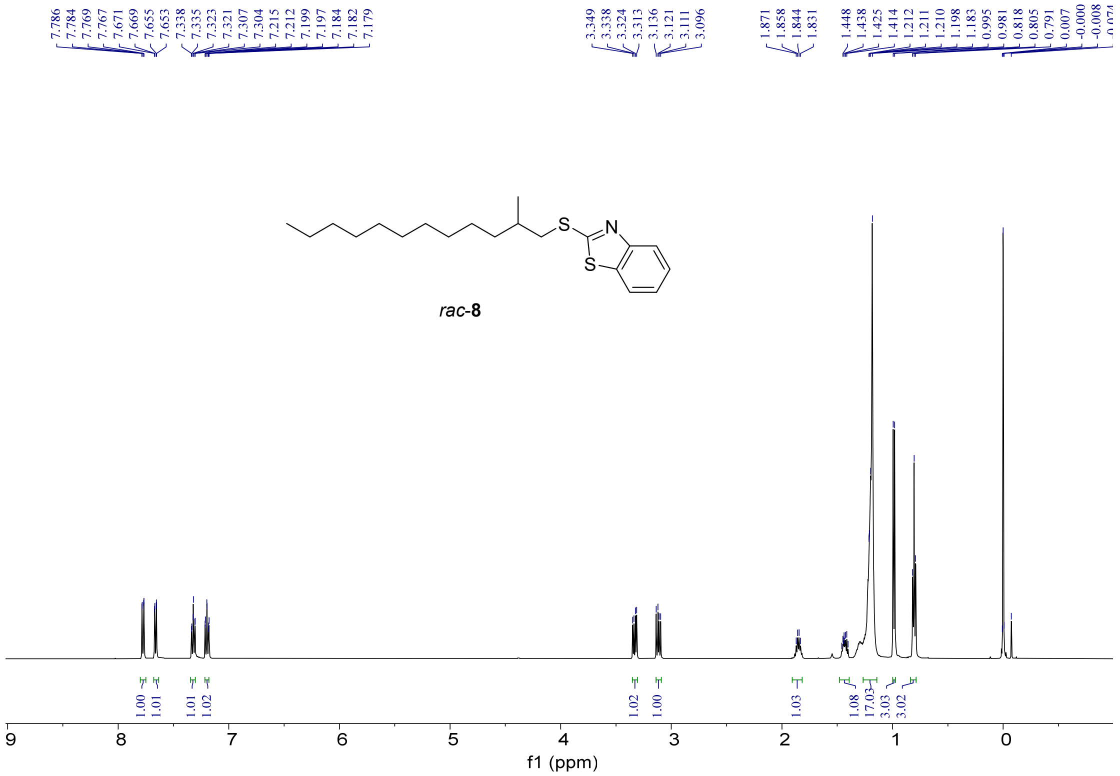

Figure S17. ^{1}H NMR Spectrum of 2-((2-methyldodecyl)thio)benzo[d]thiazole (*rac*-**8**) (500 MHz, $CDCl_3$)

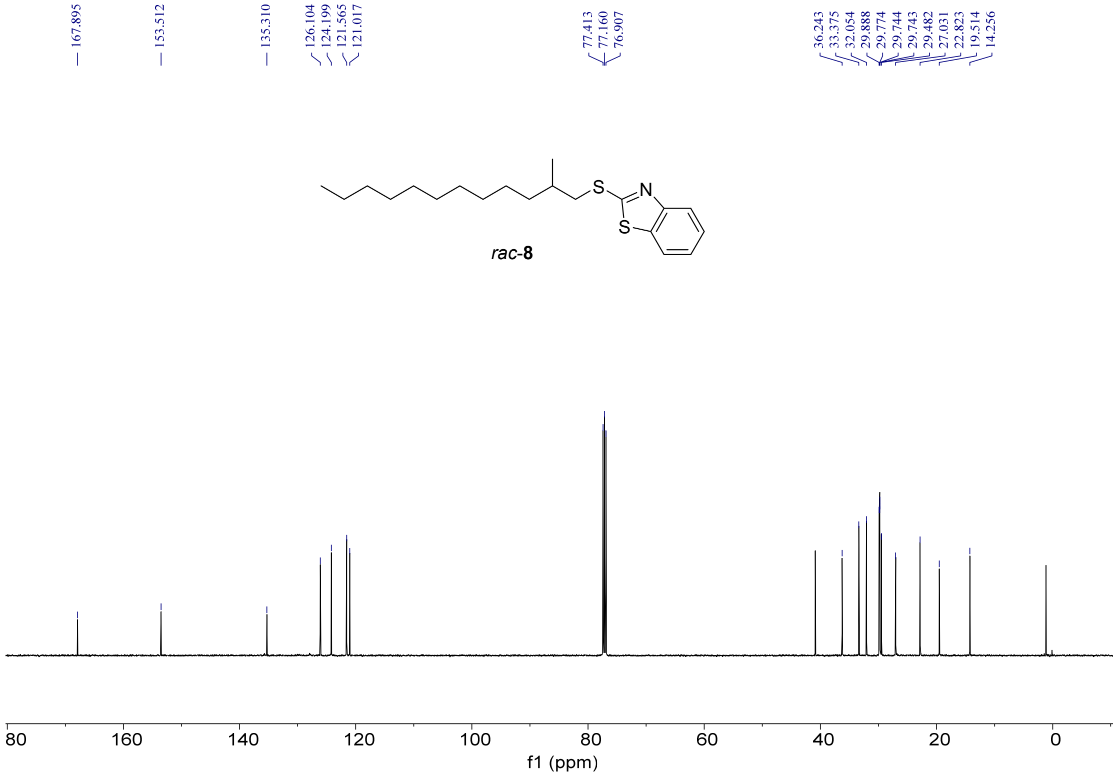

Figure S18. ^{13}C NMR Spectrum of 2-((2-methyldodecyl)thio)benzo[d]thiazole (*rac*-**8**) (126 MHz, $CDCl_3$)

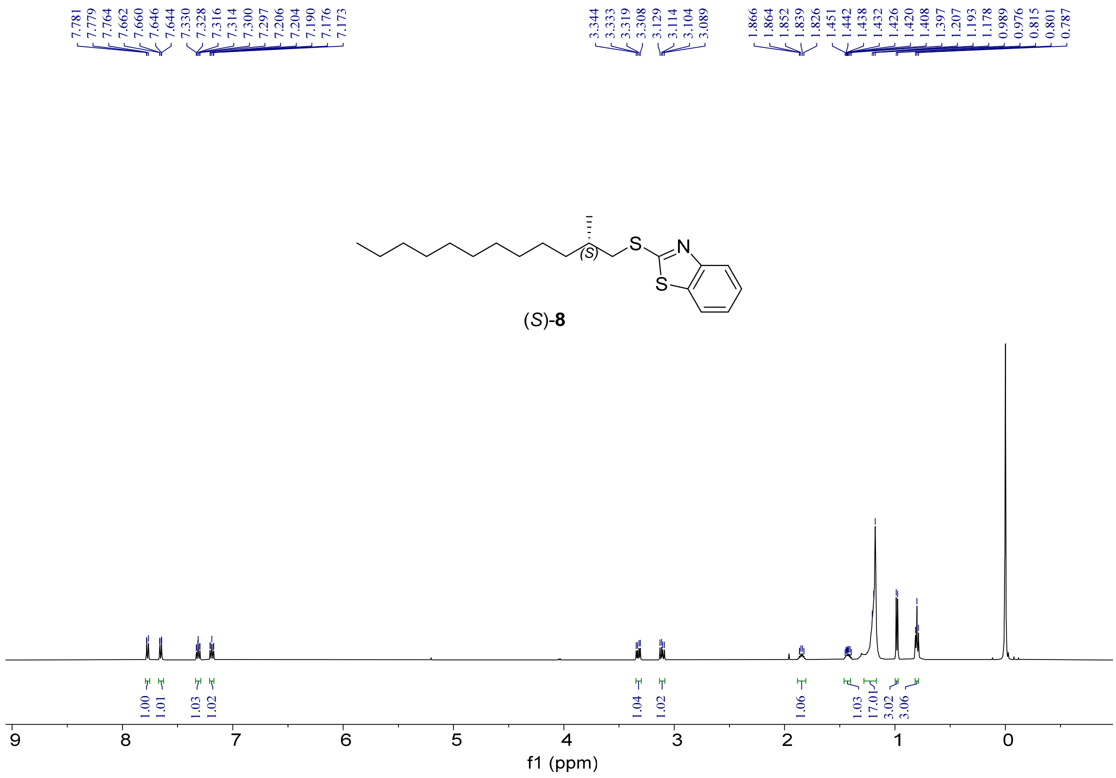

Figure S19. ^{1}H NMR Spectrum of (*S*)-2-((2-methyldodecyl)thio)benzo[d]thiazole ((*S*)-**8**) (500 MHz, $CDCl_3$)

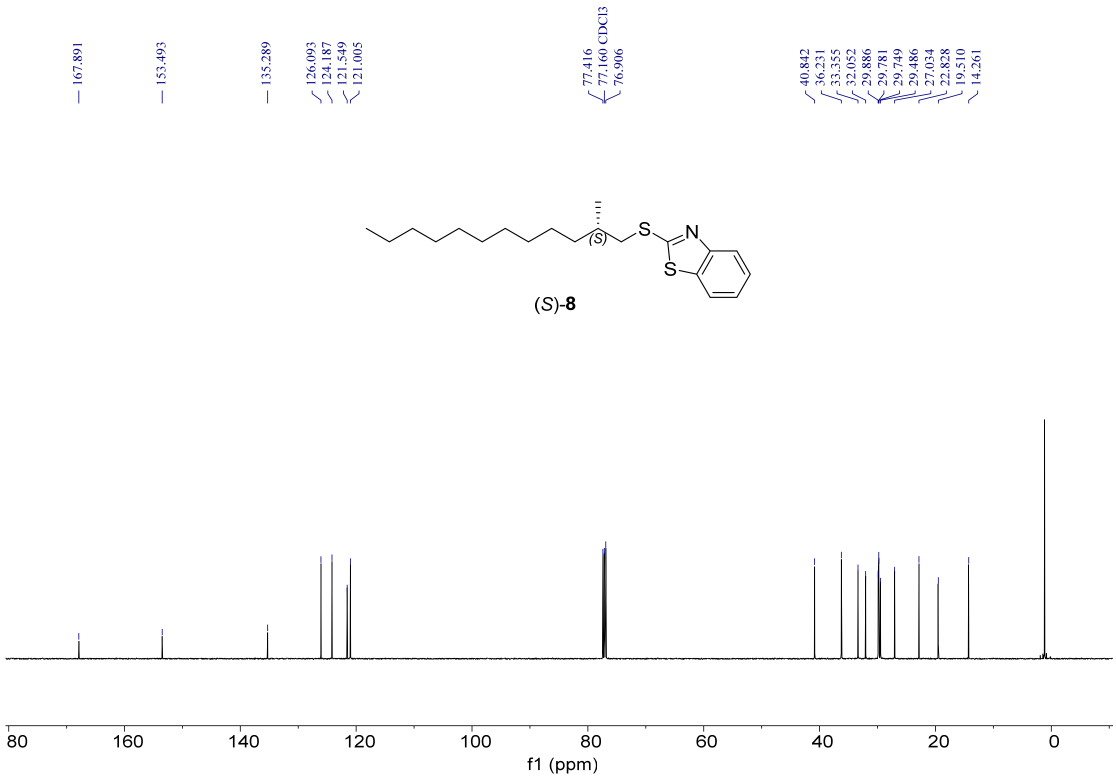

Figure S20. ^{13}C NMR Spectrum of (*S*)-2-((2-methyldodecyl)thio)benzo[d]thiazole ((*S*)-**8**) (126 MHz, $CDCl_3$)

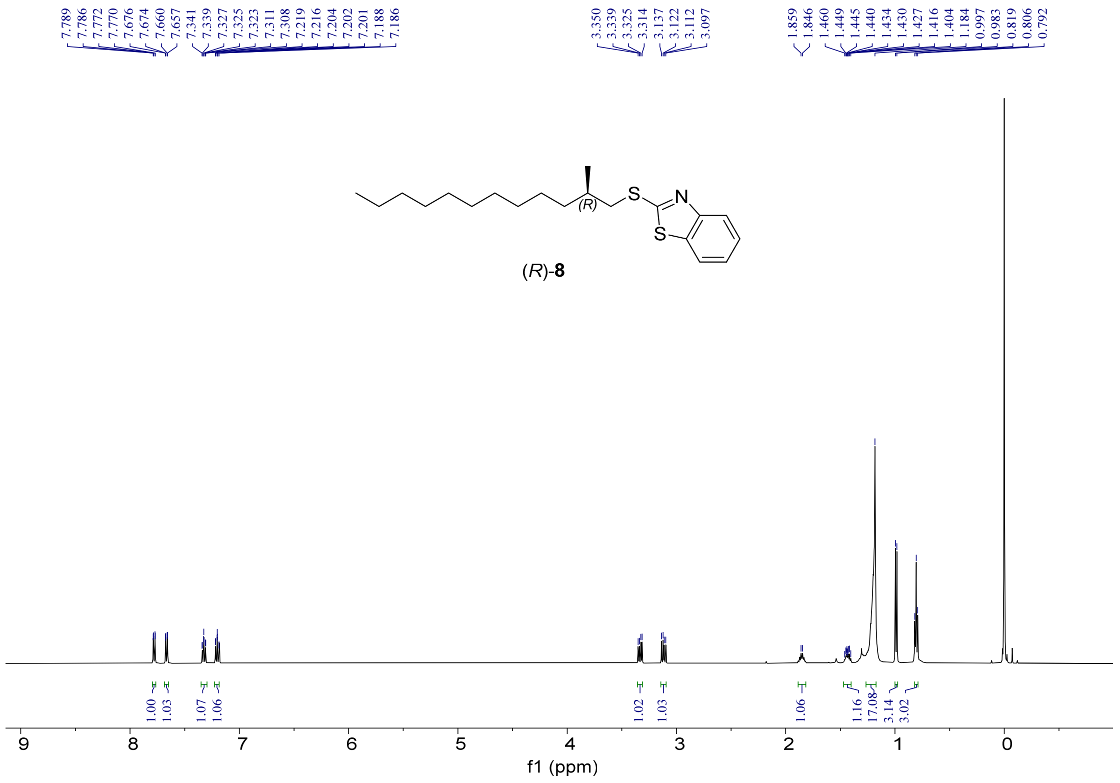

Figure S21. ^{1}H NMR Spectrum of (*R*)-2-((2-methyldodecyl)thio)benzo[d]thiazole ((*R*)-**8**) (500 MHz, $CDCl_3$)

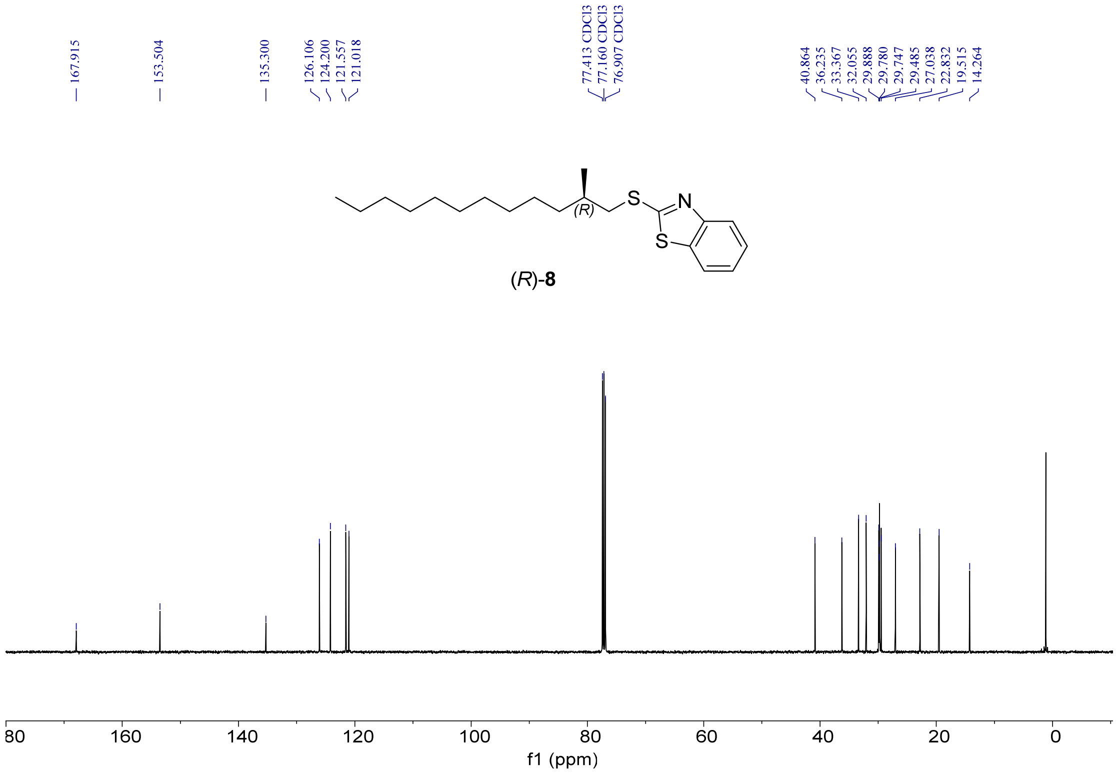

Figure S22. ^{13}C NMR Spectrum of (*R*)-2-((2-methyldodecyl)thio)benzo[d]thiazole ((*R*)-**8**) (126 MHz, $CDCl_3$)

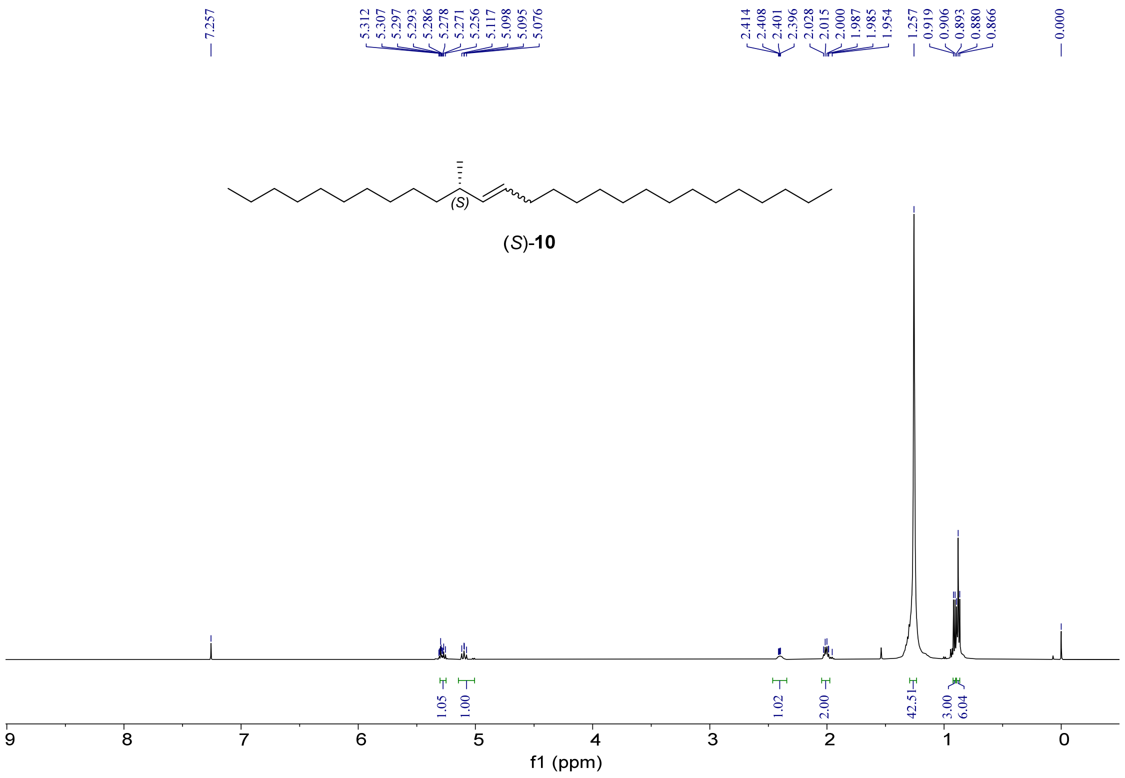

Figure S23. ^{1}H NMR Spectrum of (*S*)-11-methylheptacos-9-ene ((*S*)-**10**) (500 MHz, $CDCl_3$)

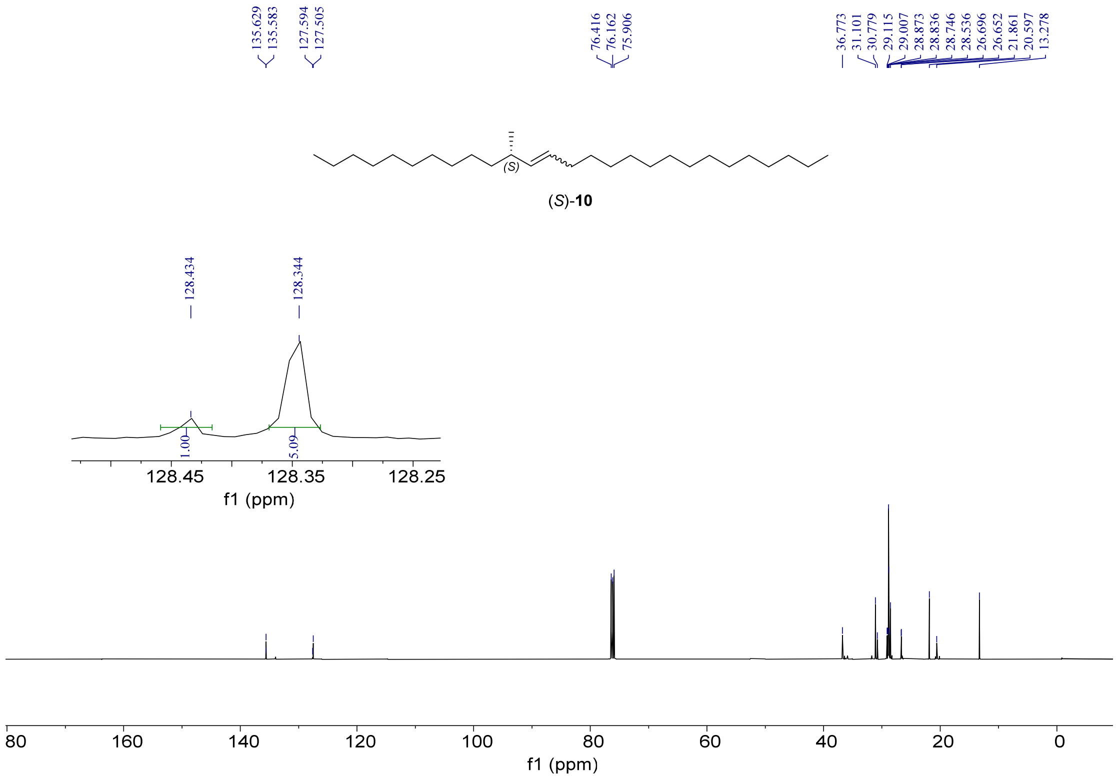

Figure S24. ^{13}C NMR Spectrum of (*S*)-11-methylheptacos-9-ene ((*S*)-**10**) (126 MHz, $CDCl_3$)

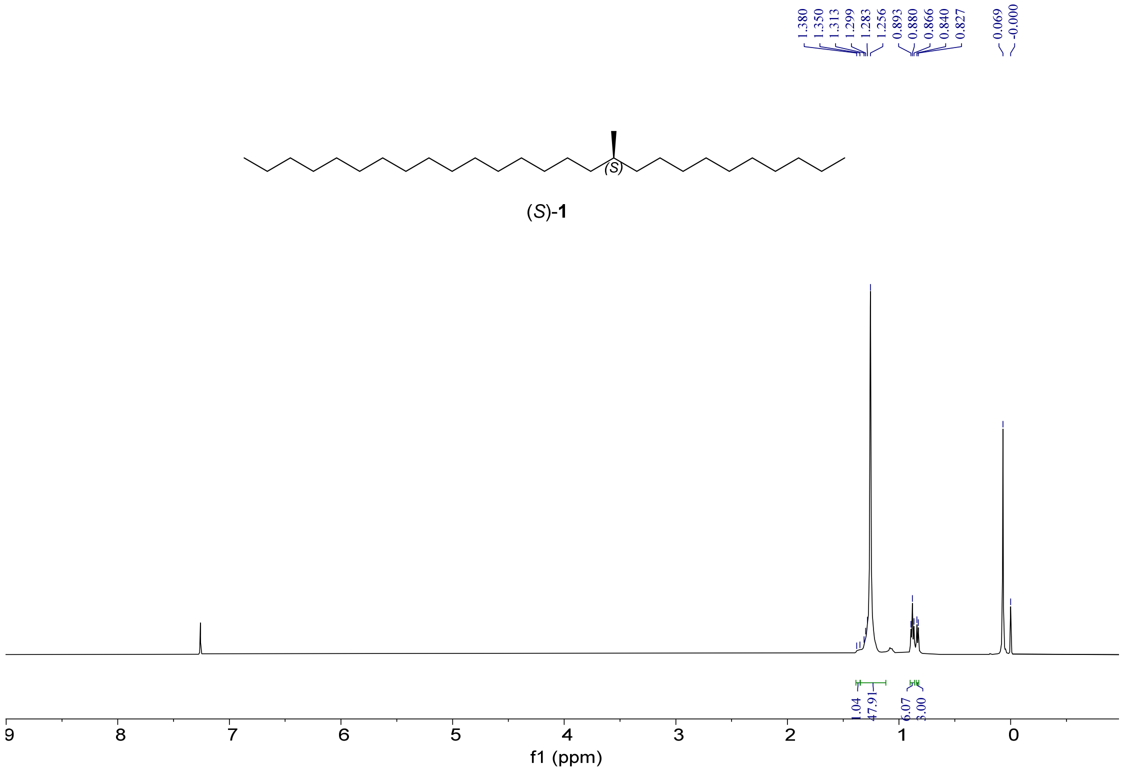

Figure S25. ^{1}H NMR Spectrum of (*S*)-11-methylheptacosane ((*S*)-**1**) (500 MHz, $CDCl_3$)

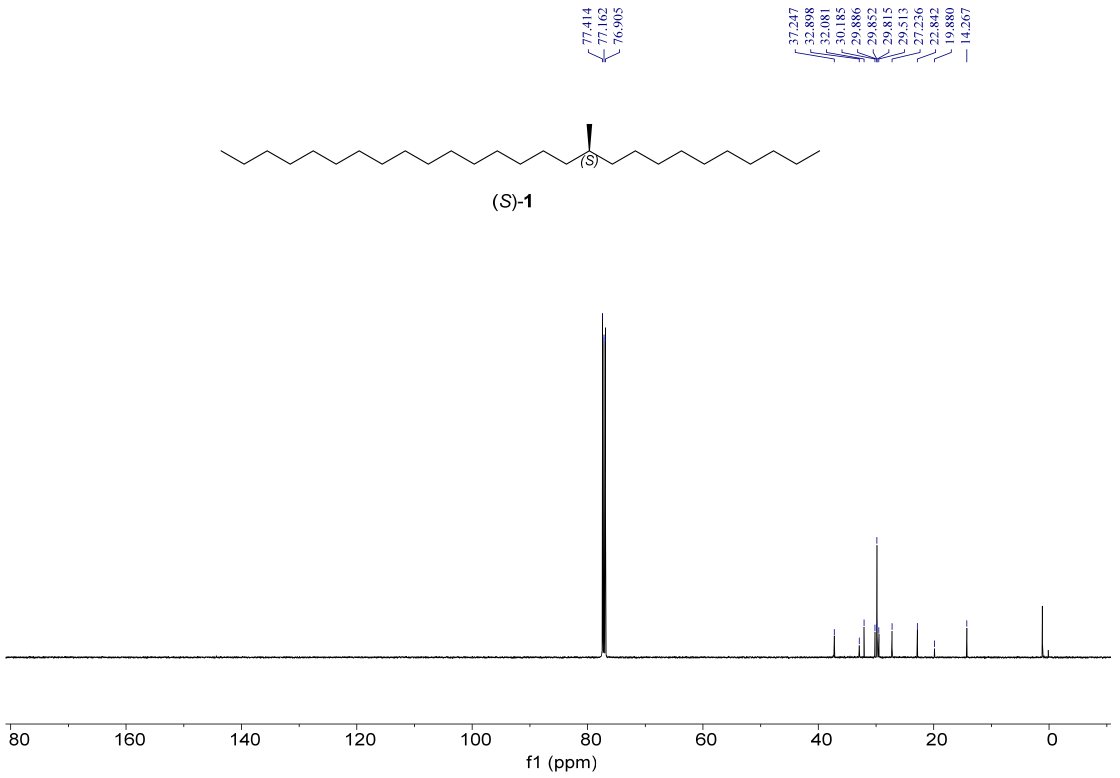

Figure S26. ^{13}C NMR Spectrum of (*S*)-11-methylheptacosane ((*S*)-**1**) (126 MHz, $CDCl_3$)

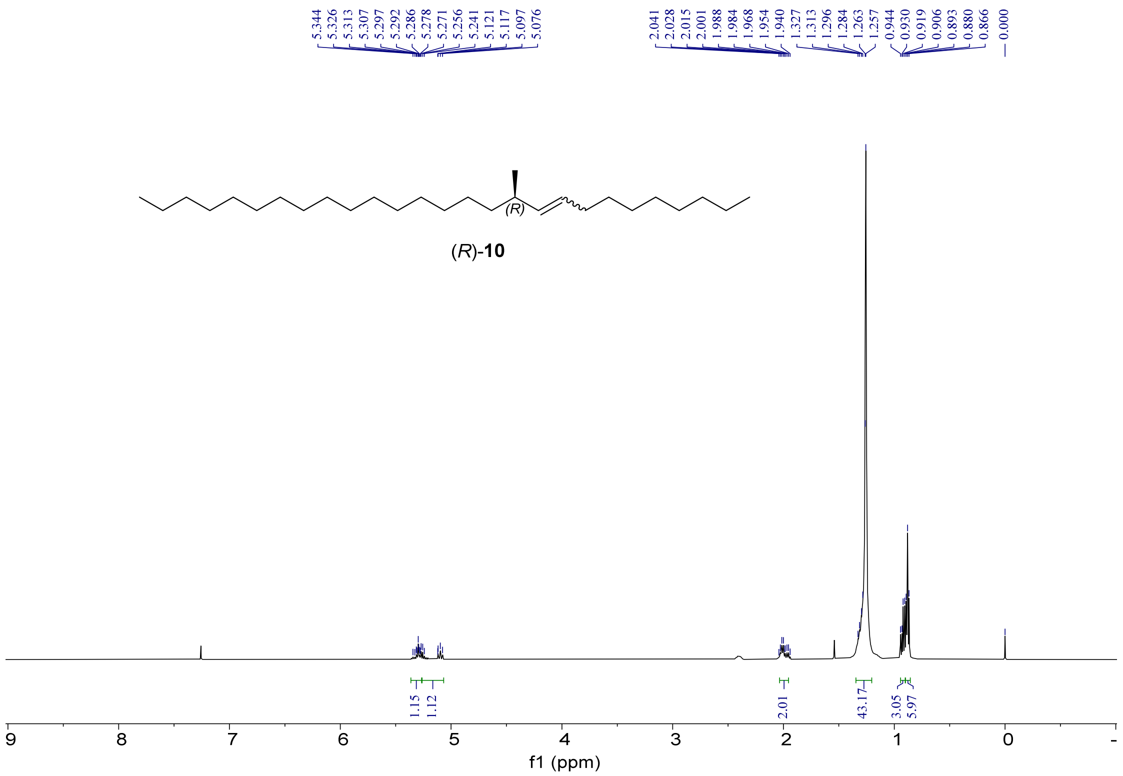

Figure S27. ^{1}H NMR Spectrum of (*R*)-11-methylheptacos-9-ene ((*R*)-**10**) (500 MHz, $CDCl_3$)

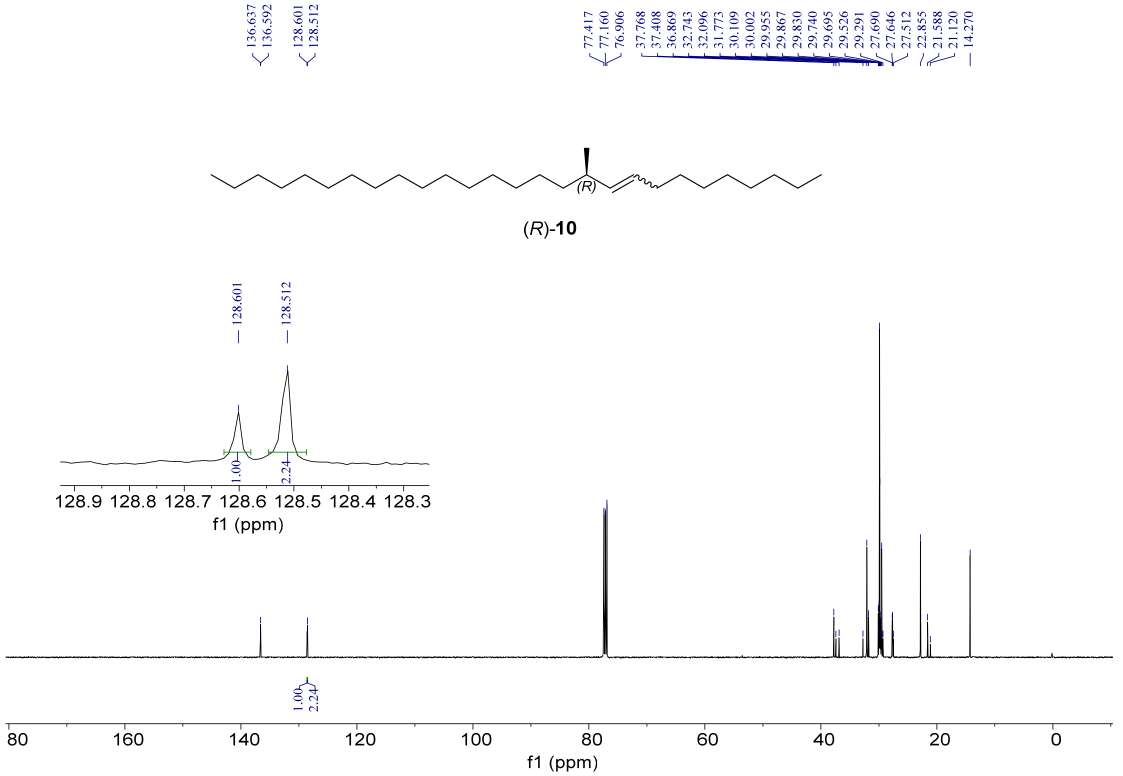

Figure S28. ^{13}C NMR Spectrum of (*R*)-11-methylheptacos-9-ene ((*R*)-**10**) (126 MHz, $CDCl_3$)

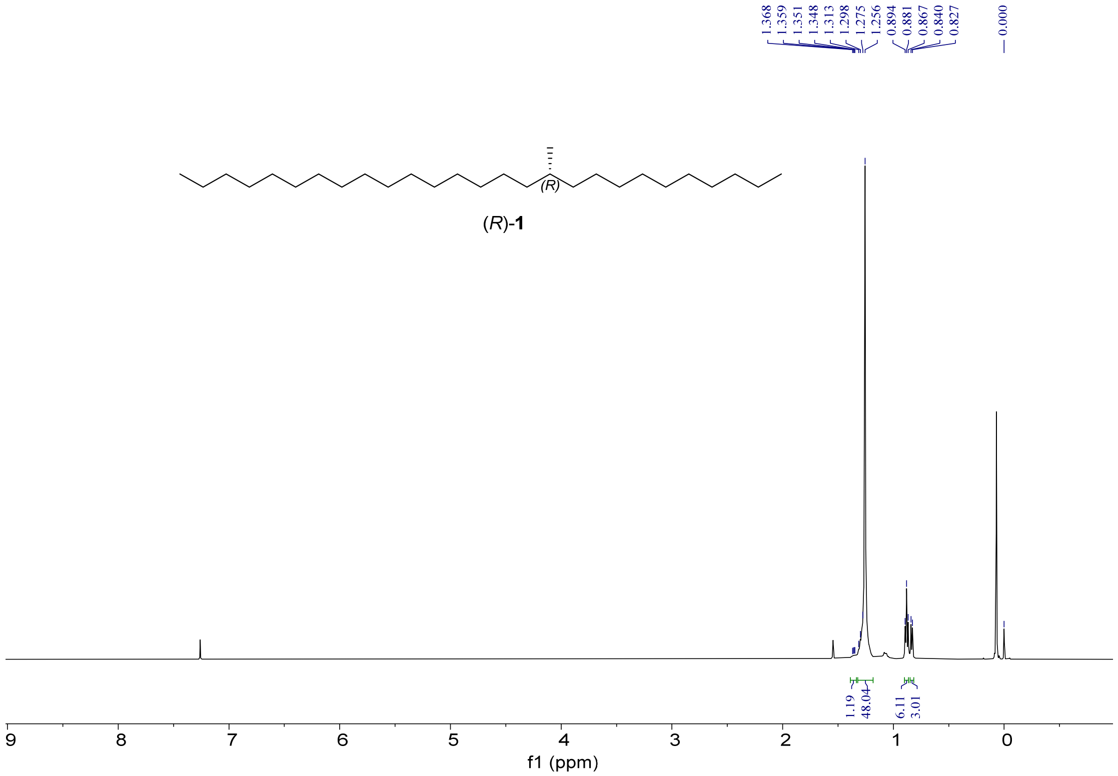

Figure S29. ^{1}H NMR Spectrum of (*R*)-11-methylheptacosane ((*R*)-**1**) (500 MHz, $CDCl_3$)

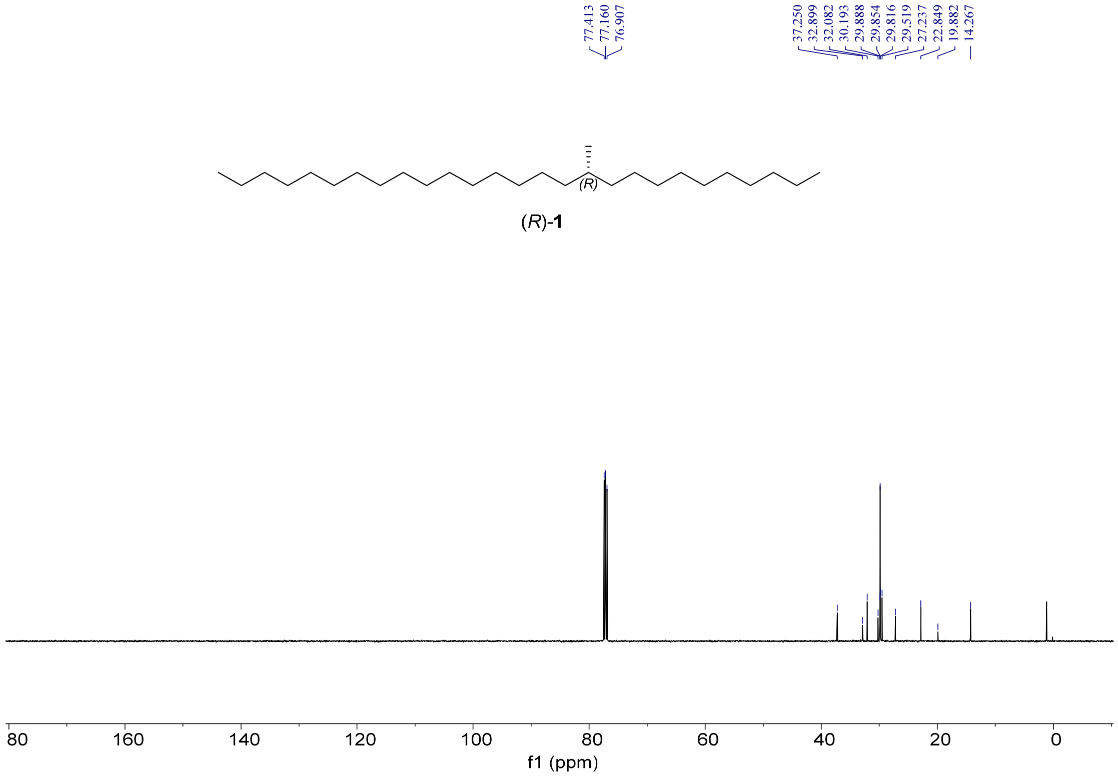

Figure S30. ^{13}C NMR Spectrum of (*R*)-11-methylheptacosane ((*R*)-**1**) (126 MHz, $CDCl_3$)

2. HPLC Chromatography of the Compounds

Figure S31. HPLC Chromatography of racemic 2-((2-methyldodecyl)thio)benzo[d]thiazole (*rac*-**8**) (Daicel Chiralcel OD-H column; *n*-hexane/*i*-propanol = 99:1, 0.7 mL/min, 254 nm)

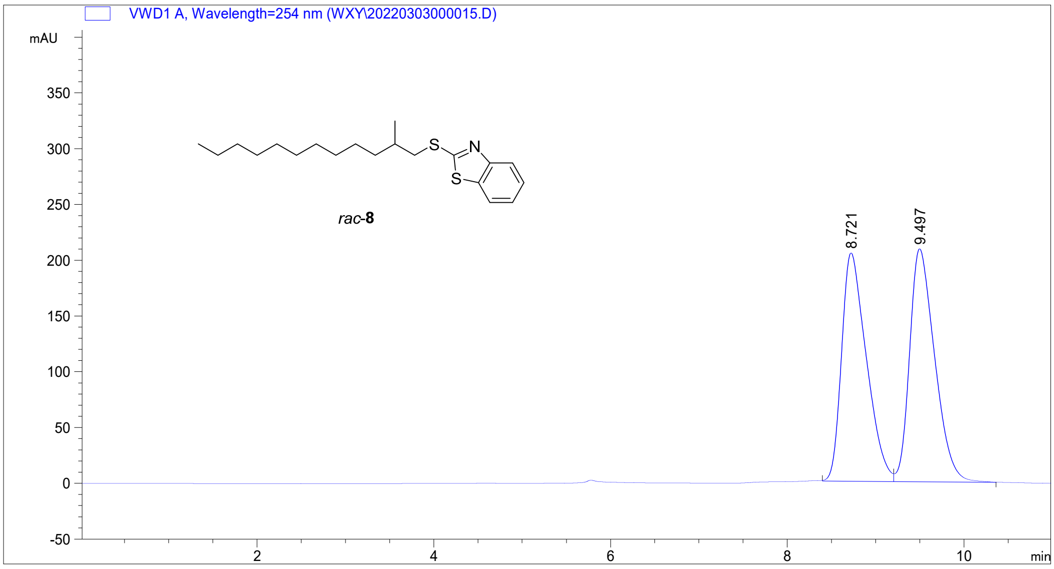

Peak	RetTime	Type	Width	Area	Height	Area
#	[min]		[min]	mAU*s	[mAU]	%
1	8.721	BV	0.2900	3992.59229	204.86427	49.1413
2	9.497	VB	0.3005	4132.12256	209.07364	50.8587
Totals:				8124.71484	413.93791	

Figure S32. HPLC Chromatography of (*S*)-2-((2-methyldodecyl)thio)benzo[d]thiazole ((*S*)-**8**) (Daicel Chiralcel OD-H column; *n*-hexane/*i*-propanol = 99:1, 0.7 mL/min, 254 nm)

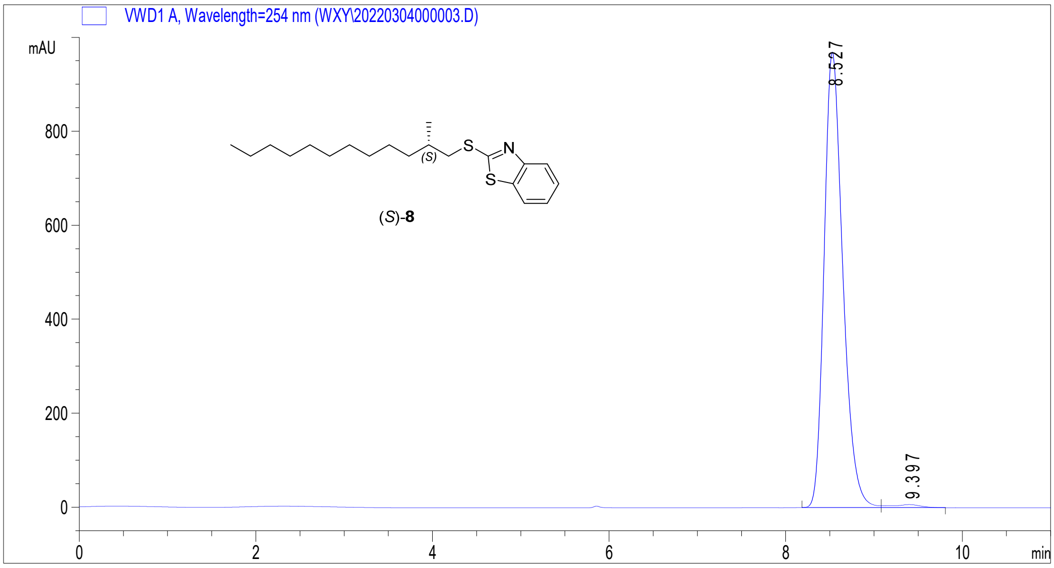

Peak	RetTime	Type	Width	Area	Height	Area
#	[min]		[min]	mAU*s	[mAU]	%
1	8.527	BV	0.2240	1.42628e4	969.64209	98.8850
2	9.397	VB	0.3263	160.82082	6.88656	1.1150
Totals:				1.44236e4	976.52865	

Figure S33. HPLC Chromatography of (*R*)-2-((2-methyldodecyl)thio)benzo[d]thiazole ((*R*)-**8**) (Daicel Chiralcel OD-H column; *n*-hexane/*i*-propanol = 99:1, 0.7 mL/min, 254 nm)

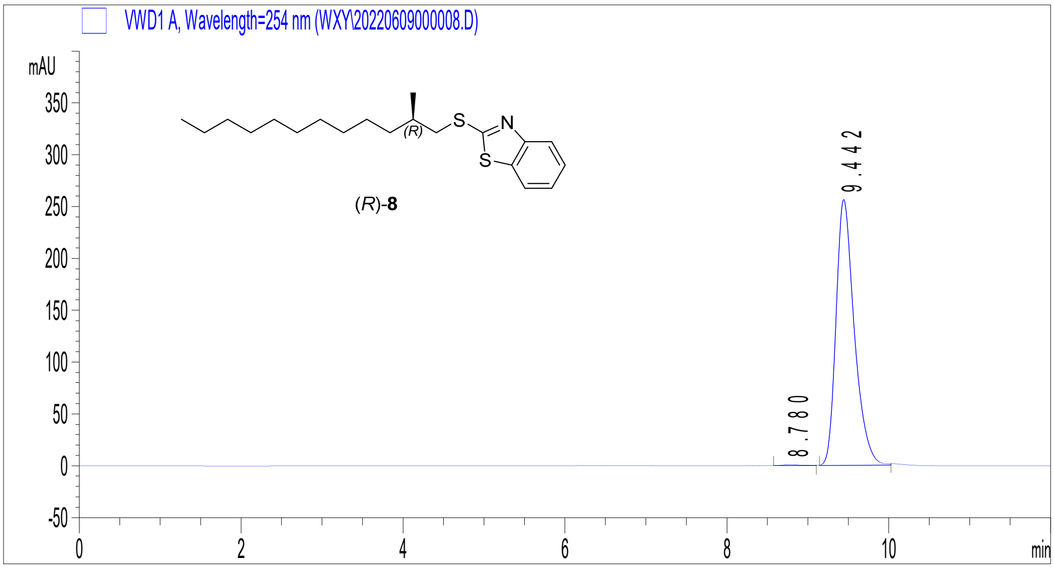

Peak #	RetTime [min]	Type	Width [min]	Area mAU*s	Height [mAU]	Area %
1	8.780	BB	0.2182	16.30239	1.11812	0.4028
2	9.442	BB	0.2372	4031.32129	256.58481	99.5972
Totals:				4047.62368	257.70293	
